# Phylogenetic simulation of promoter evolution: estimation and modeling of binding site turnover events and assessment of their impact on alignment tools

**DOI:** 10.1186/gb-2007-8-10-r225

**Published:** 2007-10-24

**Authors:** Weichun Huang, Joseph R Nevins, Uwe Ohler

**Affiliations:** 1 Institute for Genome Sciences and Policy, Duke University, Durham, NC 27708, USA; 2 Current address: Department of Biology, Boston College, Chestnut Hill, MA 02467, USA

## Abstract

**Background:**

The phenomenon of functional site turnover has important implications for the study of regulatory region evolution, such as for promoter sequence alignments and transcription factor binding site (TFBS) identification. At present, it remains difficult to estimate TFBS turnover rates on real genomic sequences, as reliable mappings of functional sites across related species are often not available. As an alternative, we introduce a flexible new simulation system, Phylogenetic Simulation of Promoter Evolution (PSPE), designed to study functional site turnovers in regulatory sequences.

**Results:**

Using PSPE, we study replacement turnover rates of different individual TFBSs and simple modules of two sites under neutral evolutionary functional constraints. We find that TFBS replacement turnover can happen rapidly in promoters, and turnover rates vary significantly among different TFBSs and modules. We assess the influence of different constraints such as insertion/deletion rate and translocation distances. Complementing the simulations, we give simple but effective mathematical models for TFBS turnover rate prediction. As one important application of PSPE, we also present a first systematic evaluation of multiple sequence aligners regarding their capability of detecting TFBSs in promoters with site turnovers.

**Conclusion:**

PSPE allows researchers for the first time to investigate TFBS replacement turnovers in promoters systematically. The assessment of alignment tools points out the limitations of current approaches to identify TFBSs in non-coding sequences, where turnover events of functional sites may happen frequently, and where we are interested in assessing the similarity on the functional level. PSPE is freely available at the authors' website.

## Abstract 2

Phylogenetic simulation of promoter evolution were used to analyze functional site turnover in regulatory sequences.

## Background

Transcription regulation is a central component in the control of gene expression. Identification of functional *cis*-elements in promoter regions, a key to understanding gene regulation, has turned out to be a difficult task thus far. With the increasing availability of genome sequences, phylogenetic footprinting appeared to offer a very promising approach for identifying *cis*-elements [1,2]. One essential assumption of phylogenetic footprinting is sequence conservation of functionally homologous genes. While such an assumption has been frequently found to be true for protein encoding sequences, there is no straightforward relationship of conservation between sequence and function for non-protein-coding regulatory sequences [3,4].

Compared to protein-coding regions, transcriptional promoter regions are subject to much less stringent selection and have higher nucleotide substitution rates, where short transcription factor binding sites can easily turn over and be replaced by new ones arising from random mutations [5,6]. In many cases, the function of a regulatory sequence may, however, remain well conserved despite substantial sequence changes. One of the best-studied examples is the *even-skipped *enhancer system *S2E *of *Drosophila *species, which is highly conserved at the functional level (for example, maintaining a high similarity of expression pattern) but substantially diverged at the sequence level. Such sequence divergence includes large insertions and deletions between different sites, substitutions within sites, and gains and losses of sites. Several experimental studies suggested that compensatory mutations in the *even-skipped *enhancer region are the key to maintain the functionality of the enhancer in evolution [7-9]. Estimates of transcription factor binding site (TFBS) turnover rates rank as high as 32-40% between human and rodent species [6], and can also happen at transcription start sites (TSSs) of orthologous genes [10], albeit at a lower frequency. The phenomenon of TFBS turnovers in regulatory regions suggest that any phylogenetic footprinting methods based on a simple trace of the evolution of nucleotides can be highly effective in some cases, but are unlikely to be able to identify all functionally important elements in regulatory genomic sequences, particularly in distantly related species. In this sense, a major improvement in TFBS identification will rely on a better understanding of evolutionary mechanisms regarding TFBS turnover events.

While TFBS turnover has been known for a long time, it has not become a widely studied topic until recently, when the availability of related genome sequences made it amenable to systematic studies [11-13]. With our currently limited knowledge about their structure and functional constraints, it is much more challenging to study the evolution of regulatory sequences than of protein-coding sequences. Most published experimental studies have been conducted on a gene-by-gene and element-by-element basis, and computational studies on real data are severely limited by the available functional site mapping data. In the absence of real biological data, computational simulation may provide the best way to study TFBS evolution and turnover in a systematic way. A pioneering simulation of TFBS evolution estimated the expected time for new binding sites to arise from point mutations in promoter regions, where binding sites were represented by simple consensus sequences, and promoters were evolved under a neutral evolution model [5]. A recent study examined the expected time for a new site to evolve and become fixed in a population by positive selection, where the authors considered effective population size and used position weight matrices (PWMs) to model TFBSs [14]. The study found that the existence and location of pre-sites of functional sites could be major factors determining the expected time and location of newly evolved sites, while the relative position of sites had little impact on the final location of new functional sites.

The above simulation studies explicitly assume that the functions encoded in regulatory regions evolve and change with the change in sequences. There are, however, many cases like the evolution of the *even-skipped *enhancer mentioned above, in which the regulatory sequence changes but functions (that is, the resulting expression patterns) appear unchanged. Frequently, such genes are involved in crucial developmental processes and, therefore, subject to stringent functional constraints [15-18]. Our study thus investigates how a promoter evolves under the neutral scenario of functional maintenance in 'status quo', that is, with little or no change in the presence and strength of functional elements. Specifically, we address the expected replacement turnover rate (RTR) of TFBSs in promoter sequences in relation to evolutionary distance, insertion/deletion (InDel) rate, and restricted translocation distance of TFBSs. In accordance with previous work, our study suggests that replacement turnover of TFBSs can happen quickly in evolution and varies significantly among different TFBSs, but can be predicted using simple mathematical models.

TFBS turnover phenomena in promoter sequences raise the important question about the ability of current multiple sequence alignment (MSA) tools to identify TFBSs in comparative genomics studies. Comparative evaluations of alignment tools have been conducted previously, but usually in conjunction with a newly developed tool [19-22] and with only few attempts at a comprehensive or systematic evaluation of different tools [23-26]. However, little has been done regarding a performance evaluation of MSA tools for the task of aligning non-coding genomic sequences, largely due to lack of good benchmark datasets of real sequences. As a result, tool performance assessment on genomic sequences was often based on indirect measures, such as an alignment of putative conserved non-coding regions, functional sites [21], or exon regions [27].

**Figure 1:**
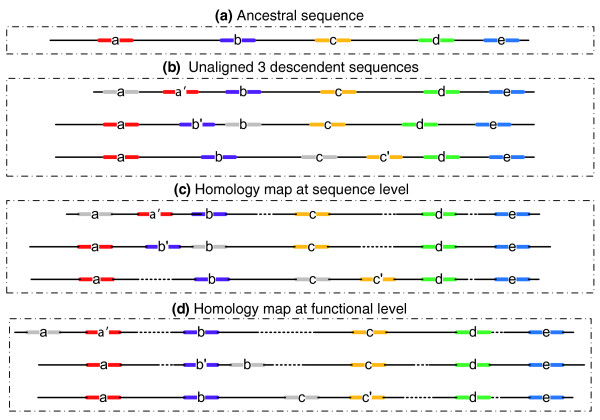
Illustration of the difference between a sequence homology map and a functional homology map. **(a) **An ancestral promoter sequence with five functional sites. **(b) **Three unaligned descendent sequences derived from the ancestral promoter sequence. In the first descendent sequence, the old site *a *was functionally replaced by the new site *a' *because of evolutionary sequence changes. Similar replacement turnovers occurred at site *b *in the second and site *c *in the third descendent sequence, respectively. The three TFBS pairs *a*-*a'*, *b*-*b'*, and *c*-*c' *are homologous at the functional level but not at the sequence level. **(c) **Alignment of the three descendent sequences based on sequence base-pair homology. **(d) **Alignment of the three descendent sequences based on their homology at the functional level. The figure illustrates cases in which it is easier to identify functional elements *a*(*a'*), *b*(*b'*), and *c*(*c'*) and to predict gene functions from the homology map at the functional level rather than at the sequence level.

Simulation provides an effective way to circumvent the problem of lack of data. Simulation data generated *in silico *make it possible to evaluate tool performance on direct measures of alignment accuracy. For example, a careful work on tool benchmarking was based on simulated *Drosophila *non-coding sequences, in which the authors compared the accuracy, sensitivity and specificity of several tools for pair-wise alignment [28]. A recent simulation study by the same group examined the limitations of several MSA tools for TFBS identification and divergence distance estimation in aligning non-coding sequences, where TFBSs may be gained or lost in neutral evolution [29]. However, these evaluation studies implicitly assumed a strong correlation between conservation at the functional and sequence level, and assessed tools on their ability to align homologous base pairs, that is, the alignment accuracy of bases evolved from the same site in the common ancestral sequences. Different from protein coding sequences, however, many recent studies of non-coding sequence evolution suggest that frequently there is only a weak correlation between conservation at the functional level and sequence level among non-coding orthologous sequences [1,3,6-8,10] (see Figure 1 for an example of homology at the functional level and sequence level).

Uncovering TFBSs in promoter sequences by cross-species comparison has so far been successful in some cases, but most approaches rely on alignments that are pre-computed on the whole genome. It is an open issue how appropriate these strategies are for non-coding alignments. Taking advantage of our Phylogenetic Simulation of Promoter Evolution (PSPE) simulation tool, we assess the performance of commonly used MSA algorithms for aligning TFBS in orthologous promoter sequences, where the function of a promoter (that is, an ensemble of binding sites under constraints) is maintained, but TFBS replacement turnovers are allowed to occur. Different from previous studies that assessed tool performance with respect to their ability to align homologous bases, we thus focus on assessing tool performance by their ability to align functional sites that are homologous at the functional level but may not be homologous at the sequence level. To our knowledge, no such assessment of MSA tool performance from the viewpoint of functional homology, that is, alignment of functional elements in the presence of re-arrangements and turnovers, has been carried out. Our findings can thus serve as useful references for alignment tool selection in comparative genomics and provide insights for the improvement of non-coding multiple sequence alignment.

## Results

### Simulation system

We designed a new computational system, PSPE, specifically to perform simulations of regulatory sequence evolution, such as promoter sequences. Different from other programs for sequence evolution simulation, which frequently use different evolutionary models for functional and non-functional sites, PSPE imposes a variety of functional constraints and validates at discrete intervals that these constraints are maintained. Such functional constraints include GC content, presence and strength of functional sites, location and copy number restrictions on functional sites, and space constraints between different functional sites. Depending on the specification of these constraints, turnover events are thus possible, as functional sites are not generally tied to a specific location in the sequence.

**Figure 2:**
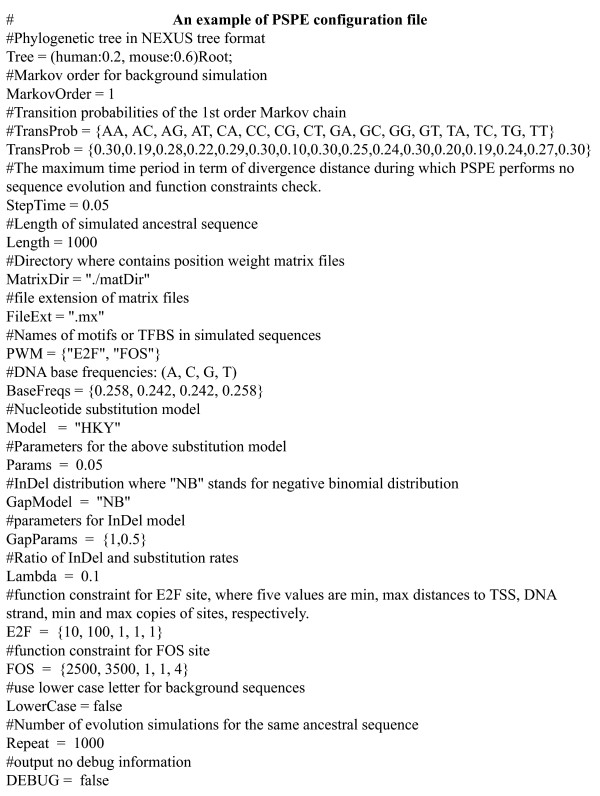
An example of a PSPE configuration file. In the configuration file, parameter names and their corresponding values are always separated by '='. The comment lines start with '#'.

PSPE reads a set of simulation parameters from a single configuration file (Figure 2). The root sequence for simulation can be provided by the user or generated by PSPE, according to user-specified length, a background Markov model, and functional constraints. PSPE can generate different random evolutionary trees by simulating evolution distances (branch length) with an exponential model, and the number of descendent sequences (number of branches from a parent node) by a Poisson process. While binary trees are commonly used in phylogenetic studies, PSPE can generate different tree structures with either a fixed or a random number of branches from the root or internal node. Given a phylogenetic tree and a sequence at its root, PSPE can use one of many commonly used DNA substitution models as well as different InDel models to simulate sequence evolution, subject to defined functional constraints, such as GC content, functional site locations and interactions of functional sites. By default, PSPE reports the alignment of the simulated sequences, as well as the sequences themselves and the locations of functional sites in each sequence. PSPE also has the capability to simulate replicates from the same tree and same root sequence, which is essential for quantitative evolution simulations.

### TFBS replacement turnover rate estimation

In this study, a functional TFBS in a descendent sequence corresponds to the original TFBS if its sequence can be traced back to the TFBS sequence in the ancestor; otherwise, the TFBS is regarded as a new one. A TFBS replacement event is therefore defined as an event in which an original TFBS is replaced by a new TFBS of the same type through any two or more events (destruction of the old site and creation of the new one), including point mutations, insertions and deletions. The RTR is defined as the probability of a functional TFBS in an ancestral sequence to be replaced by a newly evolved one in the descendent sequence. We estimate TFBS RTR as the proportion of descendent sequences in which the TFBS is replaced at least once in the evolution process from an ancestral sequence. For example, assuming that we simulate *M *different descendent sequences from the same ancestral sequence, and we observe replacement turnover of the TFBS in *m *descendent sequences, then the estimate of RTR is *m*/*M*. In the following, we report the mean RTR averaged over different ancestor sequences, that is:

$$R\hat{T}R=\frac{1}{K}\sum_{i=1}^{K}\frac{m_{i}}{M_{i}}$$

where K is the number of different ancestral sequences, *M*_*i *_is the number of all descendent sequences of the *i*^th ^ancestral sequence, and *m*_*i *_is the number of descendent sequences in which the TFBSs of interest have been subjected to replacement turnover. We also report the median values, as the distributions of RTRs are not necessarily approximate to the normal distribution.

Using PSPE for sequence evolution simulation, we are able to study the replacement turnover rate of functional conserved TFBSs in the evolution process of promoter sequences. In a complicated evolution process, many different events can occur at a TFBS, including point mutation, deletion, insertion, translocation, duplication and replacement. Our study here focuses only on TFBS replacement turnover in a simple 'status quo' scenario, assuming that all TFBSs in the sequences are essential to maintain proper gene expression levels and are thus functionally conserved in all descendent sequences. All functionally conserved TFBS are, however, allowed to be translocated to neighboring regions or replaced by newly evolved sites within a given restricted space. As ancestral sequences, we use either real or simulated human promoter sequences.

As the main transcription factor for this study, we used the well-known cell-cycle regulator E2F, and investigated two additional factors, Myc and NFκB, to validate our model for estimating TFBS replacement rates. Both E2F and Myc are important transcription regulators of cell cycle progression, DNA replication, and apoptosis [30-33]. In some cases, E2F and Myc form a complex to regulate gene expression in a combinatorial fashion [34,35]. NFκB is a family of ubiquitously expressed transcription factors involved in both the onset and the resolution of inflammation. NFκB is also widely believed to govern the expression of many genes for stress response, intercellular communications, cellular proliferation and apoptosis [36-38]. To simulate ancestral sequences containing binding sites of these transcription factors, we used their positional weight matrix models in the JASPAR database [39]. Binding sites in real human promoters known to be regulated by E2F were based on computational prediction (see Materials and methods). The simulated background promoter sequences were generated from a third order Markov model trained on 25,088 annotated human promoter sequences. We used the HKY85 model [40] to simulate nucleotide substitution, a geometric distribution for the size of sequence InDel events, and a gamma distribution and invariant rate (Γ+I) for modeling heterogeneity of substitution rates. The HKY85 model does not assume equal base frequencies and can account for the difference between transitions and transversions with one parameter. Sequence evolution was then additionally subject to diverse functional constraints related to the specific characteristics of transcriptional regulatory regions (Table 1). While many different factors may have significant impact on the RTR of a TFBS, we mainly focused on three important and interesting factors: evolution divergence distance, InDel rate, and restricted translocation distance.

**Table 1:** PSPE parameters for simulating sequence evolution

Original ancestral sequences	Human non-coding region
Sequence length	500 bp
Base frequencies	A = 0.215, C = 0.287, G = 0.285, T = 0.214
Substitution model	HKY85
Transition:transversion ratio	20:1
Point substitution:InDel ratio	10:1
InDel model	Geometric distribution (*p *= 0.5)
Heterogeneity of substitution rate	Gamma (1.0) + Iota (0.1)
Range of GC content	(45%, 70%)
Evolution distance per step	0.05 substitution per site

### Evolution of individual binding sites

**Figure 3:**
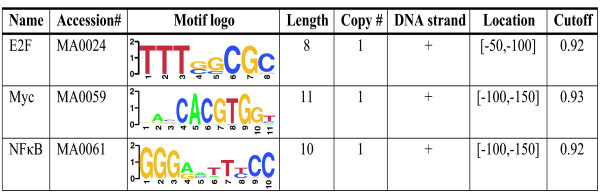
TFBSs used in the evolution simulation. PWMs of these TFBSs are taken from JASPAR [39], and their accession numbers are listed in the second column. The height of an individual letter in the motif logo represents the information content of each position in a motif. The motif logo plots were created by WebLogo [82]. The functional constraints on individual TFBSs used in the simulation are given.

We first studied the effect of divergence distance on the RTR of E2F sites (Figure 3). With increasing evolutionary divergence, we expect the RTR of a TFBS to increase, so the question is how fast and in what pattern the RTR increases along with the divergence distance. To answer this question, we estimated the RTR of a TFBS within a new descendent sequence, evolved from an ancestral sequence at 15 different divergent distances from 0.01 to 5.0, measured by the number of substitutions per site (see Materials and methods). At each of the different distances, we simulated 1,000 ancestor sequences and 1,000 descendent sequences from each ancestral sequence. In the simulation, E2F binding sites in ancestral and descendent sequences were subject to the same functional constraints (Figure 3), such that each simulated sequence had one and only one functional E2F site. As a consequence, E2F replacement could occur only at the time when the loss of the original functional site was accompanied by the creation of a new functional site. This requirement is likely to lead to conservative estimates of turnover rates.

**Figure 4:**
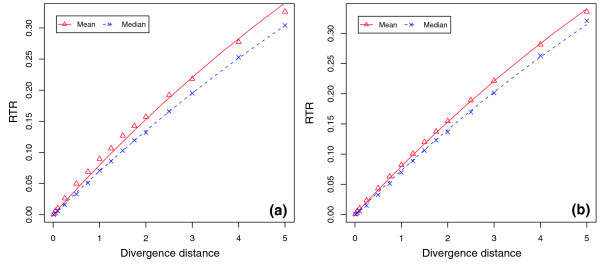
Exponential relationship between E2F replacement turnover rate and sequence divergence distance. The x-axis is the evolution divergence measured by the number of substitutions per site, and the y-axis is the RTR of an E2F site in a descendent sequence. The points are values observed from simulation, and lines are values predicted by the exponential model given in equation 2. **(a) **E2F replacement turnover rates observed in an evolution simulation starting from simulated ancestral promoter sequences, where λ is 0.0832 and 0.0724 for fitting the mean and median, respectively. **(b) **E2F replacement turnover rates observed in an evolution simulation starting from real human promoter sequences, where λ is 0.0833 and 0.0755 for fitting the mean and median, respectively.

Initial results showed that the RTR of E2F significantly increased as the divergence distance increased (Figure 4a). The change of RTR was faster at short divergence distances (number of substitutions per site <1) than at large divergence distances (number of substitutions per site >3). Based on the assumption that the number of E2F replacement events during any evolution time interval follows a Poisson distribution, we further analyzed the relationship between RTR and sequence divergence distance. Assuming that replacement turnover events occur at a Poisson rate *λ*, the probability of no replacement in a time interval *t *measured by number of substitutions per site is:

$$\mathrm{Pr}(N=0)=\frac{e^{-\lambda t}{\left(\lambda t\right)}^{0}}{0!}=e^{-\lambda t}$$

Therefore, the probability of at least one replacement turnover, or expected RTR, of a TFBS in a time interval *t *is:

*RTR *= Pr(*N *≥ 1) = 1 - Pr(*N *= 0) = 1 - *e*^-λ*t*^

which corresponds to the cumulative density function of an exponential distribution with mean 1/*λ*.

We fitted the observed E2F RTR data with this exponential model and estimated the model parameter *λ*. This simple exponential model fitted well with the RTR of E2F observed in our simulation (Figure 4a), where the model parameter λ was 0.0832 and 0.0724 for fitting the mean and median of the observed RTR, respectively. In other words, the average probability for a replacement turnover event of an E2F binding site was 8.3% at a divergence distance of one substitution per site, suggesting the potential of substantial E2F turnover.

To verify the RTR of E2F estimated on simulated promoter sequences, we repeated the experiment using real promoter sequences of human genes as ancestral sequences, known to be under E2F regulation from wet-lab experiments [41,42]. Among 127 E2F regulated genes confirmed by ChIP-chip experiments [42], we were able to select 11 genes, each having one and only one E2F binding site in the upstream region of 500 base pairs (bp) from its transcription start site (see Materials and methods; see Additional data file 1 for details of the 11 genes). Most of the 11 genes are well known to be under regulation of E2F, especially *CDC6*, for which the location of the E2F binding site and functional activity of E2F have been characterized [43-45]. Real promoter sequences would presumably give us a more realistic estimate of RTR of E2F sites than starting from simulated background sequences. One such potential difference is that real promoter sequences may contain remnants or 'ghosts' of previously functional binding sites accumulated during evolution, which could become functional again by a small number of sequence changes, which would thus result in higher turnover rates.

Starting with the real promoter sequences, we ran essentially the same simulation as the simulated promoter sequences above (Table 1), with the minor difference of using a different restricted location of E2F sites for each promoter, as the actual E2F locations were different. We kept, however, the same restricted distance for translocation of E2F sites as those in simulated promoter sequence (50 bp centered on the ancestral site). Since we had a limited number of real promoters, we simulated 10,000 descendent sequences from each ancestral promoter instead of 1,000 descendents as above. The RTRs of E2F sites estimated in this way were highly consistent with those using simulated ancestral sequences across different divergence distances. As a result, the exponential model given in equation 2 fitted well with the observed RTRs (Figure 4b), where the model parameter λ was 0.0833 and 0.0755 for fitting mean and median values, respectively. Both λ values were indeed slightly higher than the corresponding ones starting from simulated ancestral sequences (Table 2), but such small differences may easily be caused by other factors (for example, different locations of E2F sites).

**Table 2:** Estimated exponential rates associated with replacement turnovers of different TFBSs

TFBS	Promoter	λ_mean_	λ_median_
E2F	Simulated	0.0832	0.0724
E2F	Real	0.0833	0.0756
MycMax	Simulated	0.2200	0.2293
NFκB	Simulated	0.1032	0.0918

The probability of replacement turnover in evolution can be predicted by an exponential cumulative distribution function of divergence distance: *RTR *= 1 - *Exp *(-*λ *× *d*). *λ*_*mean *_and *λ*_*median *_are estimated rates for mean and median values, respectively.

To validate the good fit of estimated turnover rates with a simple exponential model, we performed similar independent simulation studies for the additional TFBSs of Myc and NFκB. Both Myc and NFκB have palindromic binding sites with a length of 11 and 10 bases, respectively. Myc sites have more conserved positions in the center region, consisting of mixed A/T and G/C nucleotides, whereas NFκB has highly conserved positions at the two sides, consisting of mostly G/C nucleotides (Figure 3). Overall, Myc sites are the most degenerate among the three TFBSs. These differences in information content and sequence composition may lead to different RTRs. It was instructive to see how these factors affected the RTR, and whether the exponential model provided as good a fit for these other TFBS as well. For each TFBS, we again simulated 1,000 ancestral promoter sequences, and for each ancestral promoter sequence, we simulated 1,000 descendent sequences at each of 15 divergence distances as above. We also used the same substitution and InDel models for the sequence evolution (Table 1). For the purpose of comparison, we imposed the same location and copy number constraints on both TFBSs as specified in Figure 3.

**Figure 5:**
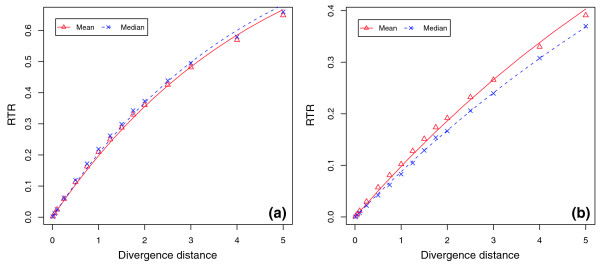
RTRs of Myc and NFΚB in simulated promoter sequences. The x-axis denotes evolutionary divergence measured by the number of substitutions per site, and the y-axis denotes the RTR of a TFBS in a descendent sequence. The figure shows that the predicted values (lines) from the exponential model given in equation 2 fit well with observed RTR values (points) from an evolution simulation of **(a) **Myc and **(b) **NFΚB.

Our results indicated that the RTR of Myc was consistently more than two times higher than that of NFκB across all divergence distances (Figure 5 and Table 2) For example, the observed RTRs for Myc and NFκB were 0.219 and 0.083 at a divergence distance of 1.0, and 0.373 and 0.167 at a divergence distance of 2.0. These results suggested that differences in sequence composition had a significant impact on the RTRs of a TFBS. In this case, the sequence composition of the NFκB site, which is G/C rich at the two sides and A/T rich in the center, is more different from the background than that of Myc, for which A/T and G/C positions are almost uniformly distributed. Fitting the RTR data with our exponential model, we observed again a good fit for both TFBSs (see Table 2 for the estimated model parameters *λ*).

### Turnover rates of regulatory modules: the Myc-E2F pair

Both Myc and E2F are important transcription factors in coordinating cell-cycle regulation, and partner together to regulate some common target genes [34,35]. As a restricted space between two TFBSs, that is, to enable an effective interaction, can limit the replacement turnover of each individual TFBS, we were interested in assessing how two sites can evolve together as a regulatory module. We studied the RTR of the Myc-E2F pair in a simple scenario in which there was one and only one pair of Myc-E2F in a promoter sequence. For both E2F and Myc, we kept the location restriction relative to the TSS identical to the above studies on single sites, and studied their RTRs by simulations with and without a constraint of restricted space between them (Table 3). We performed simulations at different divergence distances as for individual sites above.

**Table 3:** Functional constraints placed on a Myc-E2F pair in promoter sequences

E2F location relative to TSS	[-50, -100]
Myc location relative to TSS	[-100, -150]
Copy number of E2F	1
Copy number of Myc	1
DNA strand of E2F site	+
DNA strand of Myc site	+
Additional space constraint between Myc and E2F sites	[50, 60]

**Figure 6:**
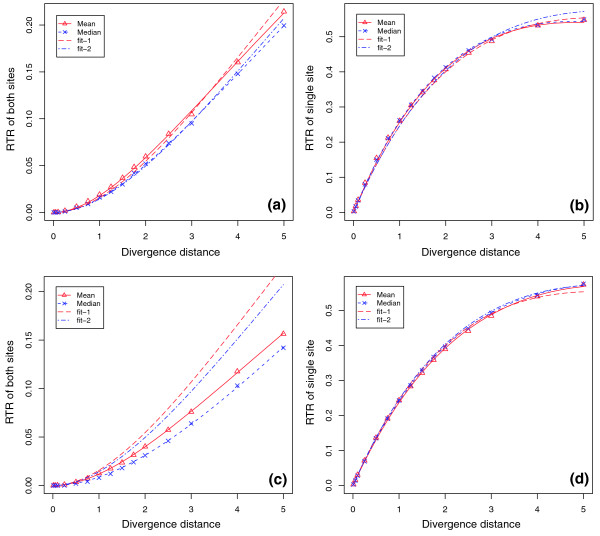
RTR of a Myc-E2F pair. We calculated the observed RTRs of Myc-E2F from simulations with and without an additional space constraint between two TFBSs, and compared the observed and expected RTRs assuming independence. The fit-1 lines are expected values based on the mean turnover rate of individual TFBSs, and the fit-2 lines are expected values based on median turnover rate of individual TFBSs. Under simulation without space constraints between the sites, the expected RTRs are close to the observed ones in both cases: **(a) **replacement turnover occurred at both Myc and E2F sites; **(b) **replacement turnover occurred at only one of two sites. Under simulation with space constraint, the expected RTRs are higher than the observed ones when **(c) **replacement turnover occurred at both Myc and E2F sites, but are close to observed ones when **(d) **replacement turnover occurred at only one of the two sites. The models based on estimates of turnover for individual sites given in equations 3 and 4 fit the observed RTR data well in those cases where no dependency between sites exists.

We calculated the observed RTRs of the Myc-E2F pair from the simulated sequences, and compared them to the expected ones assuming independent evolution of both sites. The expected RTR of both sites, defined as the probability of observing simultaneous replacement turnovers of both Myc and E2F, was estimated as the product of the individual RTRs from the simulation of single sites. The expected RTR of a single site, defined as the probability of observing a replacement turnover in only one site of the pair, was estimated from the above simulation of individual sites. Results showed that the expected RTRs were close to the observed ones in simulations without an additional space constraint between two TFBSs (Figure 6a,b), validating the independent evolution of both sites. For the simulation with additional space constraints between the pair, the observed RTRs of both sites showed significant deviation from the predicted ones assuming independent evolution, although the expected and observed RTRs of single sites were still close (Figure 6d). The significantly lower RTRs of both sites indicate that the space constraint between two sites made it less likely for them to turn over simultaneously (Figure 6c).

The small difference between the observed RTRs of the Myc-E2F pair and the expected ones assuming independence of individual TFBSs suggested that it was reasonable to describe the independent evolution of two sites within a simple predictive model. Based on this assumption, we thus described the RTR of a given TFBS pair by:

$$RTR_{pair}=(1-e^{-\lambda_{1}t})\times (1-e^{-\lambda_{2}t})$$

where *λ*_1 _and *λ*_2 _are the expected Poisson rates of replacement turnover events for TFBS 1 (E2F) and TFBS 2 (Myc). Similarly, the probability of a replacement turnover of one and only one of two TFBSs can be modeled by:

$$RTR_{one\_in\_pair}=(1-e^{-\lambda_{1}t})\times e^{-\lambda_{2}t}+e^{-\lambda_{1}t}\times (1-e^{-\lambda_{2}t})$$

We fitted the observed RTR data with both models 3 and 4. Both models fitted well with data as shown in Figure 6a,b,d, validating our assumption for the independent evolution of TFBSs. However, as the RTRs for the Myc-E2F pair in Figure 6c show, the simple models began to deviate from the simulations in more complex scenarios including dependencies between sites.

### TFBS conservation between human and mouse

Because of the moderate divergence distance between mammalian genomes, such as those of human and mouse, there is a strong interest in comparative studies of their genomes as an important way to infer gene function and gene regulation as well as their evolutionary mechanisms. While it is relatively easy to compare the coding sequences of human and mouse orthologous genes, it remains a difficult task to compare their promoter sequences, largely because they are more divergent than coding sequences. One pioneering comparative genomics study estimated that a fraction as high as 32-40% of the human functional TFBSs may not be functional in rodents, suggesting a high turnover rate of TFBSs [6]. A recent study estimated that the divergence distances of human and mouse from the last common ancestor are 0.1187 and 0.3987 substitutions per site, respectively [46]. Another study estimated the total divergence distance of human and mouse at about 0.8 substitutions per site [47]. Based on these two estimates, we here set the divergence distances of human and mouse from their last common ancestor to be 0.2 and 0.6, respectively, in terms of the number of substitutions per site in neutrally evolving regions. In this study, we simulated TFBS evolution of human and mouse from their last common ancestral species in the hope of shedding some light on the evolution of their TFBSs. Using the same three TFBSs as above, we estimated RTRs of individual TFBSs in human and mouse orthologous sequences at different InDel rates as well as at different restricted translocation distances.

#### Effect of InDel rate variation

We again simulated 1,000 ancestral promoter sequences and evolved 1,000 pairs of human and mouse descendent sequences from each ancestral sequence, but this time varying the ratio of InDel to substitution rate from 0 (that is, no InDels at all) to 0.2 (one InDel per five substitution events) at ten different steps. Except for the InDel rate, we used the same models and parameters as given in Table 1. We performed three independent simulations for the TFBSs of E2F, Myc and NFκB. The evolution of individual TFBSs was under the same functional constraints as above (Figure 3).

**Figure 7:**
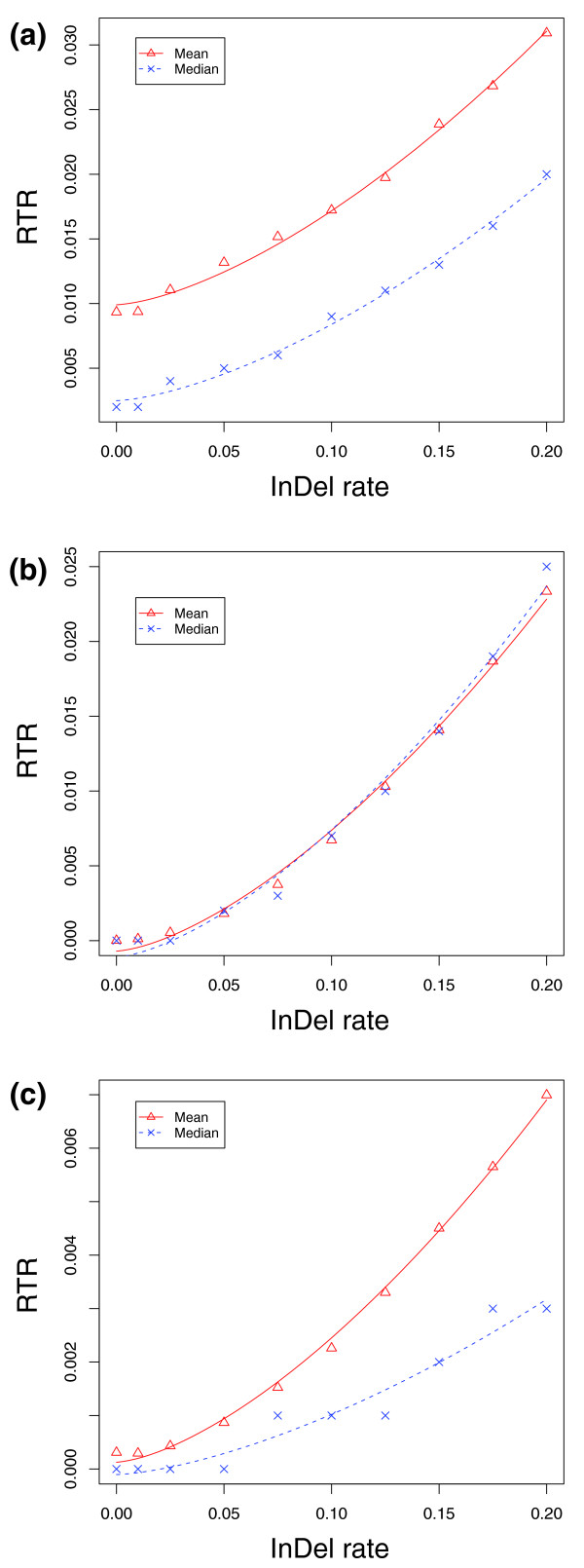
Effect of different InDel rates on TFBS RTR. The x-axis denotes the InDel rate measured by the number of InDel events per substitution events, and the y-axis shows the RTR of a TFBS in a descendent sequence. The figure shows that the exponential model given in equation 5 fits well with the observed RTR values from simulation for all three TFBSs: **(a) **E2F, **(b) **Myc, and **(c) **NFκB.

Instead of calculating the TFBS RTRs from their common ancestral sequences, we estimated the probability of observing replacement turnovers of individual TFBSs in at least one species, which we defined as the RTR between human and mouse. We found that at zero or very low InDel rates, the RTRs of Myc and NFκB between human and mouse were almost zero, whereas E2F had a low RTR (Figure 7). As expected, RTRs of all TFBSs increased as the InDel rate increased. The RTR of NFκB, however, was almost one magnitude smaller than that of either E2F or Myc, indicating a significant effect of the nucleotide composition of different TFBSs. Our analysis suggested that the TFBS RTR between human and mouse could be approximated by an exponential function of the InDel rate given by:

*Rate *= -*a *+ *b *× *e*^1.5γ^

where *a *and *b *are parameters, and γ is the InDel rate. Therefore, at a zero InDel rate (γ = 0), the base RTR is (*b *- *a*), which cannot be less than the zero, implying that *b *must be larger or equal to *a*. We found that this model fitted well with the RTR data of all three TFBSs regardless of using the mean or median value of the RTR (Figure 7). Estimates of model parameters for the individual TFBSs are given in Table 4.

**Table 4:** Estimated parameter values for the exponential model of RTR and InDel rate

	Model for mean		Model for median	
		
TFBS name	*a*	*b*	*a*	*b*
E2F	-0.216	0.226	-0.181	0.184
Myc	-0.252	0.252	-0.265	0.265
NFκB	-0.072	0.072	-0.035	0.035

Simulation results suggested that the TFBS RTR can be modeled by an exponential function of InDel rates given in equation 5. The values for parameters *a *and *b *were estimated from observed mean and median values of RTRs at different InDel rates.

#### Influence of restricted translocation distance

TFBS often have a preferred location relative to the TSS, but many TFBSs can move within a limited distance while maintaining their regulatory function. Such a restricted translocation distance relative to the TSS may have an important impact on TFBS evolution. In a final simulation, we studied how the RTR of a TFBS between human and mouse was affected by its restricted translocation distance.

**Figure 8:**
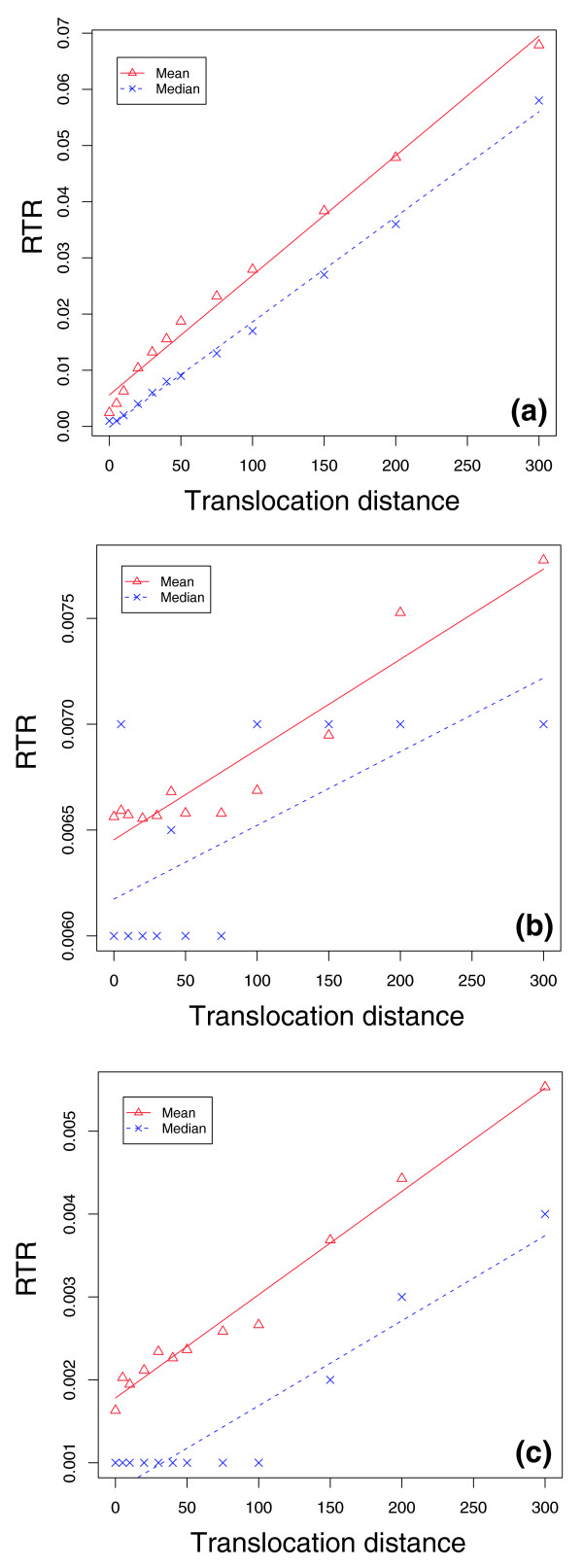
Effect of restricted translocation distance on TFBS RTR. The x-axis is the restricted translocation distance relative to the original binding site in the ancestral sequence, and the y-axis is the RTR of TFBSs. The points are the RTR observed in simulations, and lines are values predicted by the model given in equation 6: **(a) **E2F, **(b) **Myc, and **(c) **NFκB.

We simulated TFBS evolution under 10 different restricted distances of translocation ranging from 0 to 300 bp from the original location of a TFBS in ancestral sequences, where we set 20 bp as the minimum distance of a TFBS to TSS. For each maximal translocation distance, we simulated 1,000 ancestral promoter sequences and 1,000 pairs of descendent human and mouse sequences from each ancestral sequence using the models given in Table 1. We performed a separate simulation for the same three TFBSs, and estimated the RTR between human and mouse as defined above. The RTR between human and mouse increased approximately linearly with the size of the restricted translocation range (Figure 8). The means of the RTR could therefore be fitted well with a linear model given by:

$$RTR=a+c1\sqrt{\theta }\times c2\sqrt{\theta }=a+c\theta$$

where *a*, *c1*, *c2 *and *c *are model parameters, *c *is the product of *c1 *and *c2*, and θ is the restriction translocation distance of a TFBS. In this model, *c1 *and *c2 *are associated with the evolutionary distances of species one and two from their last common ancestral species. Therefore, the TFBS RTR in a single species is a linear function of the square root of its restricted translocation distance. Interestingly, while the median RTRs for E2F could also be fitted quite well with this model (Figure 6a), the fit for Myc and NFκB was less good, hinting at the strong effects that different motifs can have on some of the promoter features studied here.

#### Impact of transition/transversion ratio

To better simulate sequences of closely related species, which generally have a higher ratio between transition and transversion substitution rates than distantly related species, we used a relatively large ratio of transition to transversion (20:1) in all the above simulations. This large ratio made sense in our case, as we simulated sequence evolution in a stepwise fashion with a small divergence distance (0.05 substitutions per site) at each step. To check whether a large change in transition to transversion ratio would have significant impact on RTRs, we also ran all the above simulations at a much smaller ratio of 4:1. We used the Wilcoxon rank sum test to check whether the difference between the means of the resulting RTRs was significantly different from zero (data not shown). We found no statistically significant differences in our results (Bonferroni-corrected significance level of *P *≤ 0.05). The results suggested that our observed replacement turnovers were slow processes relative to nucleotide substitutions.

### Evaluation of alignment tools

In addition to the theoretical studies regarding turnover rates, the PSPE simulator can be used to assess the impact of the turnover phenomenon on practical applications in comparative genomics. In the following, we looked specifically at the problem of identifying functional binding sites in multiple sequence alignments. Most current alignment tools are based on the assumption that the functional sites in orthologous sequences are homologous in sequence space, that is, that they can be traced back to the same position in the ancestral genome. Replacement turnover events of functional sites in promoter sequences, however, make this assumption somewhat unrealistic, which could consequently limit the performance of a tool for aligning non-coding sequences. Our evaluation aimed to: compare different multiple sequence alignment tools for their robustness to violation of this assumption; and investigate the impact of increasing the number of species on tool performance.

**Figure 9:**
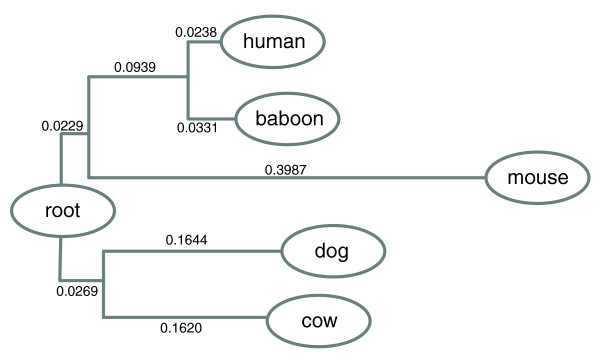
Phylogenetic tree of five mammalian genomes. The evolutionary distances shown in the tree were recently inferred from the coding region of orthologous genes [46]. In our simulation, we used the tree scaled at eight different levels relative to the evolutionary distances shown.

We evaluated a set of representative MSA tools for their performance in detecting TFBSs in several sets of orthologous sequences, generated from an underlying phylogenetic tree of five mammalian genomes (Figure 9). The rationale for using the mammalian tree topology was to achieve a realistic assessment of TFBS detection accuracy and to allow for a fair comparison between different tools. First, in most comparative genomics studies, species in comparison often have different divergence distances from their last common ancestor. Second, it is also frequently assumed that an MSA tool should work better when aligning more closely related species at the beginning stage and adding more distantly related species in later stages, especially for those based on a progressive approach. We used evolutionary distances that were recently inferred from coding regions [46], but evaluated the tree at different scale factors as it is not generally known how well these distances reflect the actual substitution rates in non-coding regions. We extended the simulation to large divergence distances to test the notion that conserved sites should be readily picked up when the surrounding sequence has sufficiently diverged. To assess the validity of our observations, we consistently evaluated tool performance with additional benchmark datasets, generated from a phylogenetic tree with a star topology in which all descendent sequences had the same evolutionary distance from their last common ancestral sequence. The evaluation results are consistent with those reported below (see Additional data file 2 for details).

We scaled the mammalian phylogenetic tree at eight different levels from 0.25 to 5, relative to the actual distances, and generated a benchmark promoter dataset at each scale level (defined as divergence scale coefficient), where each dataset contained 1,000 replicates of orthologous promoter sequences of the five species. Sequences were simulated under the HKY85 nucleotide substitution model with gamma and invariant rate (Γ+I) for modeling substitution rate heterogeneity (Table 5). In the dataset, each sequence contained exactly one functional binding site for each of the six transcription factors: Pax6, TP53, IRF2, PPARG, ROAZ, and YY1E2F. YY1E2F is a composite TFBS consisting of YY1 and E2F binding sites that reportedly interact with each other in cell cycle gene regulation [48]. Binding sites were subject to a set of functional constraints (Table 6) that were set to allow for turnover within a restricted distance, but keeping the overall order of the binding sites unchanged. Simulation allowed us to quantify the amount of turnover: how many non-aligned functional sites were due to turnover compared to 'simple' misalignments, and whether some tools would in fact be able to align functional sites despite turnover.

**Table 5:** Simulation parameters used by PSPE for generating benchmark promoter sequences

Evolution distance per step	0.05 substitution per site
Length of root sequences	3,000 bp
Background sequence model	Markov order of third
Base frequencies	A = 0.258, C = 0.242, G = 0.242, T = 0.258
Substitution model	HKY85
Transition:transversion ratio	20:1
Rate heterogeneity	Gamma (1.0) + Iota (0.1)
Range of GC content	(0.45, 0.55)
Gap model	Negative binomial distribution (1, 0.5)

**Table 6:** Functional TFBS constraints used in the promoter simulation

Name	Accession no.	Length (bp)	Strand	Location (min, max)	Copy no. (min, max)	Cutoff
YY1E2F	MA0095 (YY1) MA0024 (E2F)	13	+	(20, 30)	(1, 1)	0.90
Pax6	MA0069	14	+	(50, 70)	(1, 1)	0.90
TP53	MA0106	20	+	(360, 400)	(1, 1)	0.90
IRF2	MA0051	18	+	(420, 480)	(1, 1)	0.90
PPARG	MA0066	20	+	(2000, 2080)	(1, 1)	0.90
ROAZ	MA0116	15	+	(2100, 2200)	(1, 1)	0.90

The accession numbers in the second column are from the JASPAR database [39]. 'Location' refers to the restriction on the upstream minimum and maximum distances to transcription start site. YY1E2F is a composite TFBS created by joining the YY1 and E2F sites.

We used this dataset to assess the performance of five widely used MSA tools: CLUSTALW [49], DIALIGN [50], AVID/MAVID [19,51], LAGAN/MLAGAN [27], and MUSCLE [20]. Among the five tools, AVID/MAVID is the fastest alignment tool and uses exactly matching words as alignment seeds to speed up the alignment process, albeit at the expense of lower alignment accuracy. As an improvement, both DIALIGN and LAGAN/MLAGAN adopt non-exact word matching for finding alignment seeds, which can improve their ability to detect degenerate functional sites. DIALIGN identifies alignment seeds by finding consistent sequence segments of a fixed length between sequences, while LAGAN/MLAGAN locates alignment seeds by chaining together neighboring similar words. Both CLUSTALW and MUSCLE are primarily based on the dynamic programming algorithm. MUSCLE, however, has made significant improvements over CLUSTALW by employing anchoring techniques and a progressive refinement approach. The performance was measured as TFBS detection accuracy, defined as the proportion of nucleotides in functionally homologous TFBSs that were correctly aligned. The detection accuracy reported here is the average value over 1,000 replicates at each divergence scale level.

**Figure 10:**
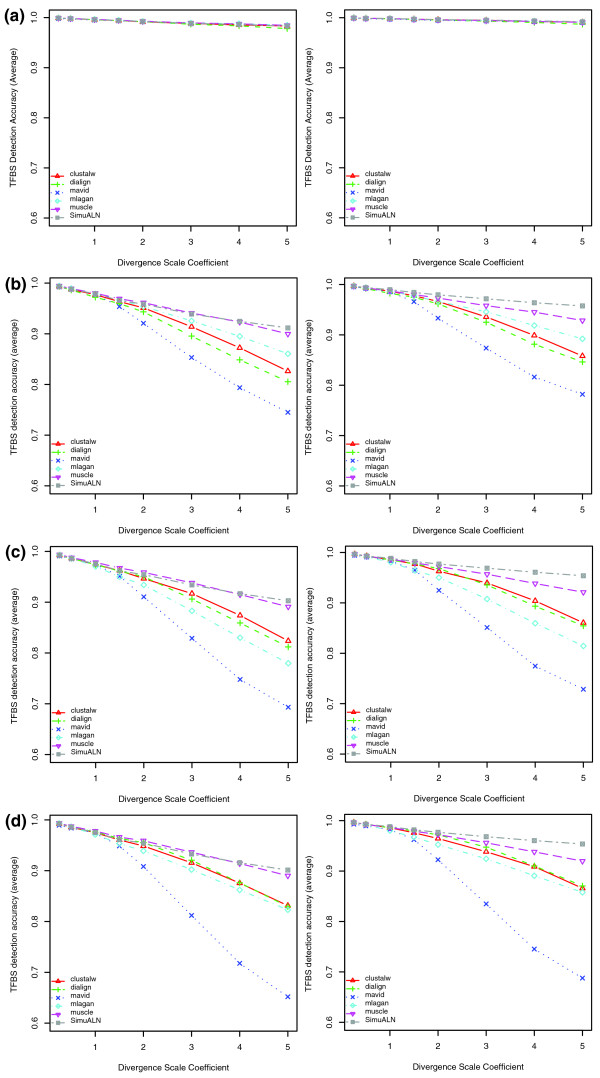
Average TFBS detection accuracy of five alignment tools. The y-axis shows the TFBS detection accuracy average of six TFBSs, and the x-axis is the divergence scale coefficient of the mammalian phylogenetic tree (Figure 9). SimuALN stands for the simulated alignment and its measure indicates the proportion of TFBS nucleotides not subject to replacement turnover in descendent sequences, and thus aligned in simulated alignments. Plots in the left panel show the overall detection accuracy of all functional TFBSs, while those in the right panel show the detection accuracy on the subset of TFBSs that had not turned over. Note that insertion and deletion events may affect parts of a binding site (these are still included in the evaluation), and that SimuALN consequently does not reach a level of one in the right panels. **(a) **Two species alignments of human and baboon. **(b) **Three species alignments of human, baboon and mouse. **(c) **Four species alignments of human, baboon, mouse, and dog. **(d) **Five species alignment.

For the two species (human and baboon) alignment, all five tools showed high detection accuracies of TFBS with no significant difference between each other (Figure 10a(1)). When adding more distant species, such as mouse, to the alignment, we found that TFBS detection accuracies of all tools were dramatically decreased, especially those of MAVID and CLUSTALW (Figure 10b(1),c(1),d(1)). Again, we observed marked differences in performance between different tools for three or more species alignments. Overall, MUSCLE had the highest detection accuracy among all tools across all divergence scale coefficients; MAVID had a slightly worse performance than all other tools; and CLUSTALW, DIALIGN and MLAGAN showed similar performance, although their relative order in performance varied with the number of species or a change of the divergence scale coefficient. As expected, the TFBS detection accuracy decreased for all tools as the divergence scale coefficient increased. PSPE also allowed us to consider only the set of sites that had not turned over, and the relative performance of tools was unchanged (Figure 10a(2),b(2),c(2),d(2)). With increasing distance, a large fraction of sites has turned over, but many of those trace back to the same ancestral nucleotides in several descendants, due to turnover before a branch in the tree or convergent evolution. These sites should thus be aligned and are counted positive in at least some of the pairwise comparisons that our metric is based on, even if they are not in the location of the original TFBS (see Additional data file 2 for more evaluations on turnover sites).

**Figure 11:**
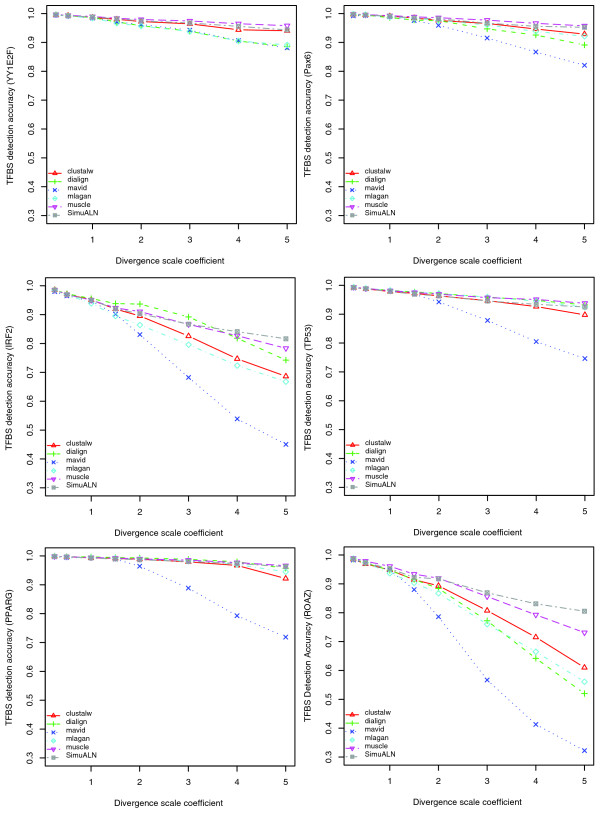
Detection accuracy of individual TFBSs on five-way mammalian alignments. All five tools perform better at detecting YY1E2F and Pax6, which have low RTRs and short restricted distance for translocation, than IRF2 and ROAZ, which have high RTR and long restricted distance for translocation. MUSCLE shows an overall better performance than the other four tools. MLAGAN performs better than DIALIGN on YY1E2F, PAX6, PPARG and ROZA, while DIALIGN shows a better performance than MLAGAN on TP53 and PPARG, which have a long restricted distance for translocation but a relatively low RTR.

The ability of a tool to detect the presence of a common TFBS varied among different TFBSs, depending on TFBS base composition, length, and restricted translocation distance, as well as the divergence scale coefficient of the phylogenetic tree. For example, Figure 11 shows that detection accuracies differed significantly among TFBSs in the alignments of the five species. In addition, the same figure shows that all tools had higher detection accuracies for TFBSs with low RTRs, such as YY1E2F and Pax6, than those with high RTRs, such as IRF2 and ROAZ. While MUSCLE showed a better performance than all other tools, CLUSTALW as the oldest tool performed slightly better than DIALIGN, MAVID, and MLAGAN in at least some cases (YY1E2F and ROAZ). Additionally, for YY1E2F, Pax6 and TP53, MUSCLE showed higher TFBS detection accuracies than the baseline of SimuALN, suggesting its capability of correctly aligning at least some TFBSs subject to turnover, that is, homologous only at the functional level. At large divergence scale coefficients, however, no tool seemed to perform well in detecting ROAZ.

**Figure 12:**
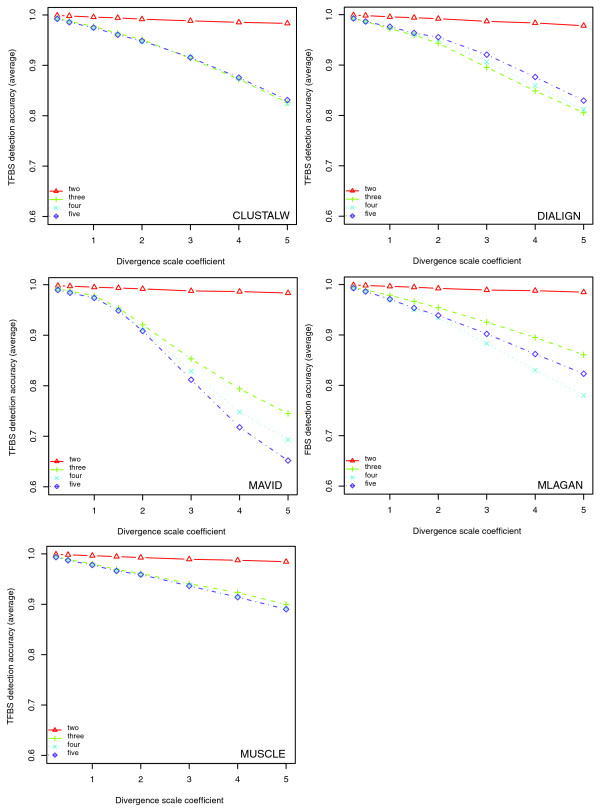
Effects of the number of aligned mammalian species on the TFBS detection accuracy. Each panel shows the performance of a tool in aligning a different number of species. Human and baboon were used for the two species alignment, mouse was added for the three species alignment, and all five species but cow were used for four species alignment. While all tools have almost the same performance for aligning the two closely related species human and baboon, MUSCLE and DIALIGN performed better than other tools in maintaining or improving performance when adding more species to the alignment.

**Figure 13:**
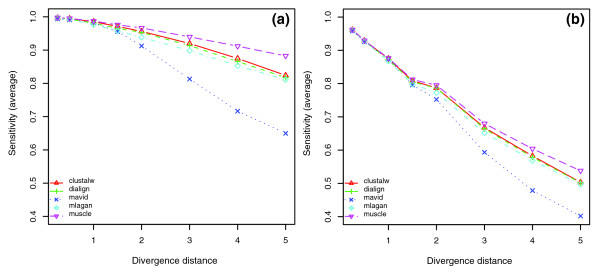
The average TFBS sensitivity of five tools in aligning TFBS in five mammalian species. **(a) **The average TFBS sensitivity of all functional TFBSs. **(b) **The average TFBS sensitivity with the subset of non-turnover sites among all TFBSs. The relative order of TFBS sensitivity for the five tools is almost the same as the order of their TFBS detection accuracy (Figure 10d).

When looking at the performance of each tool individually (Figure 12), we found that the TFBS detection accuracies of all tools decreased when adding one or more distant species to the human/baboon alignment. For alignments from three to five species, the TFBS detection accuracies of DIALIGN and MUSCLE showed little change, those of CLUSTALW and MLAGAN had a noticeable change and that of MAVID markedly decreased, especially at large divergence scale coefficients. We also compared tool performance again with respect to overall alignment sensitivity and TFBS sensitivity. We found that in terms of alignment sensitivity, MUSCLE and CLUSTALW had slightly better overall performance than the other three (data not shown). The ranks according to TFBS sensitivity were also in the same order as those according to detection accuracies, and this was also true if we considered non-turnover sites only (Figure 13).

## Discussion

In the process of evolution, selection may act directly on regulatory functions but only indirectly on gene sequences, which is supported by the experimental observations that some orthologous genes with highly conserved expression patterns have substantial divergence in their promoter sequence [7-9]. That means that functional conservation does not necessitate conservation on the sequence level. Neutral sites in promoter sequences may be free to change, and newly evolved functional sites can readily replace old ones. It is important, therefore, to understand the evolutionary mechanisms of regulatory regions in order to improve computational methods that are developed to analyze them. However, it is difficult to investigate systematically non-protein-coding evolution on real sequence data because the history of evolutionary events shaping them is largely unknown, and the map of functional sites in regulatory sequences is often incomplete and inaccurate. In many cases, there is no simple way to distinguish a site newly evolved in a replacement turnover event from one created by simple translocation of an old site. Computational simulation seems to be an effective alternative to study TFBS evolution in this case. Simulators allow us to investigate evolutionary events such as replacement turnovers of TFBS, which may significantly limit the effectiveness of phylogenetic footprinting for regulatory region identification, in an explicit way. Here, we describe a new sequence simulator to investigate the effect of different functional constraints on turnover rates, and to create a framework to evaluate multiple sequence alignment algorithms regarding their ability to detect functional elements in the presence of turnover events.

### Simulation of TFBS turnover

Our simulator PSPE is designed specifically for studying the evolution of functional sites in regulatory sequences. PSPE is not only able to use one of many common models of nucleotide substitution, but it can also apply different InDel models important for regulatory sequence simulation. In contrast to other simulators, PSPE imposes a variety of functional constraints instead of sequence constraints. Such functional constraints include GC content, presence of functional sites, strength of the binding sites, location and copy number restrictions on functional sites, and space constraints between different functional sites. All these features enable PSPE to simulate evolution of promoter sequences more realistically than other simulation programs.

Consistent with previous simulation studies [5,14], our results show that TFBS turnover can occur rapidly in promoter evolution. For example, replacement turnover events can occur at a Poisson rate as high as 0.083 for the highly constrained E2F sites even if we only allow for a small translocation distance of 50 nucleotides, and is even higher for the less constrained sites of Myc (0.22) and NFκB (0.103). Furthermore, these parameters may be relatively conservative considering that we used stringent matrix score cutoffs to avoid false hits, highly restricted locations for functional sites, a relatively low rate for transversions, and the requirement of the presence of exactly one functional site throughout. However, a high turnover rate of TFBSs can frequently be detrimental to an organism, and highly increased turnover rates may not be observed in practice, even for degenerate sites. This is supported by an additional simulation study we carried out using a lower cutoff threshold of 0.85 for functional sites, in which promoters with Myc sites had a lower RTR despite the higher chance of creating a new site at the lower cutoff. This was mainly due to our restriction of allowing only one site to be present in the promoters (see Additional data file 1 for details). Therefore, TFBS replacement turnovers in real sequences may happen more frequently than we estimated, but there is an upper limit of turnover rate for each individual TFBS imposed by the resulting changes in fitness.

Altogether, our study suggests that the TFBS RTR of a functional site between different species does not depend only on the base composition of the site and the divergence distances between species, but also on location constraints, neighboring functional sites, the InDel rate, and the GC content. While not discussed in detail, a simulation using lower GC contents showed a consistently higher or lower RTR depending on the TFBS, suggesting that the high GC content in promoter regions near the TSS is affecting the turnover rates of important functional sites (Additional data file 1). Consequently, the RTR varies not only among different functional sites and different species, but also among different instances of the same functional site upstream of different genes.

While we attempted to choose realistic model parameters and biologically meaningful functional constraints in our simulations, our estimates are certainly biased by the assumptions behind the chosen constraints, and may be substantially different from the real ones. Furthermore, the TFBS and evolution models themselves represent simplified versions of the underlying biological processes, and other factors, such as the number of replicates used in the simulation, can add some additional variation as well. We realize that the weight matrices used here as models of functional sites may not be as adequate for modeling positional dependencies as other more advanced motif models [52,53]; however, PWMs are a valid model for many biological motifs, are available in open-access databases, and are computationally more efficient than other advanced models. Computational efficiency is an important factor in simulation studies that are as large as this one.

Simple evolutionary changes within regulatory regions, such as turnover events affecting individual sites only, can be modeled effectively by Poisson events. We could show good agreement of this for a variety of binding sites and conditions, such as different translocation distances. In theory, one could derive closed-form solutions for the probability of these events, based on the sequence composition of the region and the composition and degeneracy of a binding site. However, with an increasing number of restrictions and dependencies of sites in complex regulatory modules, this becomes increasingly cumbersome and not straightforward. Figure 6c showed that these simple models begin to deviate as soon as we address the conservation on the module level instead of individual sites only.

One can easily think of a large number of additional parameters and configurations of functional sites that we did not explore. A tool such as PSPE will allow researchers to explore empirically a wider range of restrictions and complex configurations of regulatory regions in an efficient manner. Enhancers come in many different flavors, from highly restricted 'enhanceosomes' corresponding to ultra-conserved elements, to highly flexible 'billboard' enhancers allowing for many drastic sequence changes without apparent functional consequence [54]. PSPE is available to the public and we anticipate that it will be a beneficial tool for evolutionary biologists to explore the specific characteristics and evolutionary space of particular regulatory systems. Future extensions may include an adaptation for RNA regulatory regions, including specific modeling of compensatory mutations in RNA secondary structure, incorporating transposable elements, and neighbor-dependent substitution models.

### Assessment of MSA tools

During evolution, natural selection forces impose different functional constraints on protein coding and regulatory regions. The phenomenon of frequent TFBS turnovers in regulatory regions may partially explain why comparative genomics analysis, the most powerful approach so far, has met with only limited success in identifying functional sites despite the increasing availability of whole genome sequences. TFBS turnovers may also be responsible for the weak relationship between sequence conservation and functional conservation in promoter sequences, which makes the straightforward tracing of nucleotide evolution between divergent orthologous sequences meaningless with respect to their function. Our strategy of defining conservation on the level of functional constraints such as matrix score cutoffs is similar to a recent model, which defines conservation on the level of conserved binding energy [55]. In this sense, functional homology maps of regulatory regions, where mapped elements correspond to functionally equivalent sites, can be more important than strict sequence homology.

While many alignment tools have been developed so far, it is difficult to systematically evaluate and compare these tools, especially regarding their performance in aligning non-coding sequences, for which we have a limited understanding of evolutionary constraints. Studies that rigorously assess alignment tools (for example, [28]) can serve as useful reference for making more informed decisions about which tool to use for which task, and can also provide important insights or suggestions for improvement of existing algorithms. Most published evaluations of alignment algorithms were based on alignment sensitivity, specificity, and accuracy, and did not address replacement turnover of functional sites in evolution. The evaluation reported here is different: instead of trying to systematically assess all different performance aspects, we focus on one particular scenario, the capability of accurately aligning conserved TFBS in promoter sequences. Specifically, our evaluation was based on two aspects: the capability of aligning functionally homologous TFBS in promoter sequences in which TFBS replacement turnovers are allowed to occur; and the capability of increasing TFBS detection power with an increase in the number of homologous aligned species.

The five tools selected for our evaluation are representatives of many existing tools of different underlying algorithms. These differences were clearly reflected in the success of aligning TFBSs, which ranked MUSCLE at the top, AVID/MAVID at the bottom, and others in between. We purposefully chose transcription factors with long binding sites, and required strong conservation of orthologous sites (that is, a high matrix score threshold for each site). Furthermore, while our choice of constraints allowed for turnover events, it did not allow for a shuffling of sites, which none of the programs can take into account. Yet, our results suggest that the ability of existing tools to detect functionally homologous elements decreases with increasing replacement turnover rates of functional sites or, related, the sequence divergence distance. An increased divergence of the non-functional parts of the sequence does thus not necessarily help to locate individual functional binding sites, even if the sites are highly conserved and 15-20 bp long.

It is often reported that an increase in the number of species may significantly increase the power for functional site identification in comparative genomics analyses [56]. On the contrary, our evaluation results show that we should be extremely cautious at this point to assume that this is a general property of many functional DNA regions and/or tools to analyze them. With the exception of DIALIGN, the TFBS detection accuracy of all tools was either decreased or relatively unchanged in most cases. This is in fact not surprising when ones take a closer look at the approaches used for multiple sequence alignment. CLUSTALW, MAVID, and MLAGAN all use the same progressive approach for aligning multiple sequences, in which intermediate alignments from the early stages are not allowed to change in later stages. That means that the mistakes that happen in an early stage of alignment will be propagated and cannot be corrected at a later stage. Since a tool based on the progressive approach can only accumulate more mistakes when aligning more sequences, it is conceivable that its performance decreases as the number of species increases. MUSCLE employs an improved progressive approach that allows changes in the alignment of sub-groups in a recursive refinement process, which explains why MUSCLE did not show a significant decrease in performance as the number of species increased. It is conceivable, however, that the particular choice of species, and the order in which they are presented to a phylogenetic aligner, may significantly change the accuracy of these approaches. DIALIGN is the only tool surveyed here that does not use the progressive approach. Instead, it assembles the whole alignment by greedily finding all consistent segments of significant similarity from all sequences [57], which allows DIALIGN to be able to take advantage of the information from additional species. While these features of DIALIGN are interesting, there is still much room for improvement as its overall performance is no better than MUSCLE.

We want to stress that the tools in this study were not specifically developed for the alignment of non-coding regions. In fact, some design principles may be counterproductive for this task: whole genome alignments are built to provide fast comparative maps and are certainly able to detect coding conservation. The progressive aligners in our evaluation are meant to provide the phylogenetic history, that is, to compute an accurate alignment of bases that are derived from the same nucleotide in the ancestral genome. Yet, there is no doubt that many researchers currently use these tools in studies concerning gene regulatory sequences, and we hope that this evaluation provides clues about what to expect if they are used in this way. We aimed to include a representative subset of tools fast enough to perform extensive comparisons. We do not expect this to have introduced a systematic bias, but of course some recently developed aligners (for example, TBA [58], Prank [59], or Probcons [60]) may perform differently to our selected set.

The objective and systematic evaluation of alignment tools is a challenging task, in particular for an assessment on non-coding sequences whose actual functional and evolutionary mechanisms remain largely unknown. Since we simulated data under a set of specific conditions that are unlikely to represent all actual scenarios, one should carefully interpret our comparison results. For example, our study did not consider the ability of a tool to deal with very large insertions and deletions because of few large insertions/deletions in our simulated data. Furthermore, we were very conservative in our constraints, and, for example, allowed for turnover, but not for a shuffling of sites. Our results are therefore a rather optimistic estimate, and performance on real promoters with shorter sites that do not preserve their order can be expected to be significantly worse. We are also aware that the criteria for tool performance can be different in a different study depending on its objectives. Therefore, our results may not be applicable for some studies, such as the estimation of divergence distances between species. For such cases, the recent evaluation by Pollard *et al*. [29] may be a better reference.

## Conclusion

TFBS replacement turnover is an important phenomenon in the process of promoter evolution, and providing a framework to address it systematically is critical for our understanding of the mechanisms driving promoter evolution. We introduced the new simulation system PSPE, designed specifically for regulatory sequences, and allowing for functional site turnover events. PSPE is freely available at the authors' websites [61,62]. Applying PSPE in a large-scale simulation, we found that replacement turnovers could happen rapidly in promoter evolution. We also investigated different factors besides the divergence distance that significantly affect turnover rates, and describe the relationships between the RTR and different factors in simple mathematical models. Our study adds to the increasing evidence that it is important and advantageous to trace homology on the functional rather than on the sequence base-pair level in cross-species comparisons of regulatory sequences.

PSPE also provides a flexible system to generate appropriate standard test sets for alignment or motif finding algorithms, and we presented first results of this application. To our knowledge, our evaluation of MSA tools is the first one to assess their ability to detect TFBSs that are homologous on a functional level. Our evaluation of five widely used MSA tools suggests that the turnover of functional sites poses a challenge for alignment tools, even for the simplified case where the functional sites remain co-linear in orthologous sequences. While all MSA tools under consideration, especially MUSCLE, performed well in aligning functional sites at short or moderate divergence distances, they appeared to lack sufficient capability to align functional sites that have high RTRs in divergent sequences. In addition, our study suggests that the widely used progressive approach for MSA is counterproductive for the multiple alignments of homologous non-coding sequences, and that MUSCLE's improved progressive approach and DIALIGN's segment assembling approach are better suited for non-coding MSA. Some recent approaches are promising to successfully deal with the specific challenges of non-coding alignments, for example, by using available models of TFBS to 'anchor' alignments [63]. However, this still leaves us with a number of open issues on the way towards computational tools that will help us to elucidate the structure and evolution of regulatory regions.

## Materials and methods

### Background model of ancestral sequences

To generate biologically relevant ancestral sequences, we used a 3rd order Markov model to generate background sequences of ancestral promoters. We trained the background Markov model on a large real dataset of regulatory sequences extracted from the NCBI human RefSeq database (build 35). The dataset consists of 25,088 human promoter sequences each spanning a region of 500 bp immediately upstream of the transcription start sites. The base frequencies of four nucleotides were also estimated on this dataset.

### Selection of E2F regulated ancestral genes

We obtained 127 experimentally confirmed E2F regulated genes from a previous publication [42]. We removed the genes for which we were not able to extract their promoter sequences from NCBI, and extracted 500 bp long promoter sequences upstream of their annotated transcription start site. We then identified potential E2F sites in each promoter sequence using the PWM model. We removed those genes that had either zero or more than one E2F binding site based on the cutoff score of 0.92 given in Figure 3. The remaining 11 genes are given in Additional data file 1 and were used as ancestral sequences for our simulation study.

### Motif model of TFBSs

We used the PWM, a generic and widely used model for DNA motifs, to represent functional TFBSs. The PWM is generally given by a matrix with frequencies (or weights) of the four nucleotides at each position. While there are several different methods to calculate a motif score, we used a scoring function similar to the one proposed by [64] and defined by:

$$\begin{array}{l} Score=\frac{\sum_{i=1}^{w}\theta_{i}\times f_{i}^{b}}{\sum_{i=1}^{w}\theta_{i}\times \max_{b\in (A,C,G,T)}f_{i}^{b}}\\ \text{where }\theta_{\text{i}}=1+\ln4\times \sum_{b}f_{i}^{b}\ln(f_{i}^{b}),i=1...w. \end{array}$$

The function gives a normalized score from 0 to 1 for any TFBS, with 0 for the most unlikely and 1 for the most likely site. A functional binding site defined in this study is a site having a score larger than a certain cutoff threshold.

Position weight matrices of E2F, Myc, and NFκB were taken from the JASPAR database [39,65]. The functional sites in simulated ancestral sequences were generated from these PWMs. To maintain a low false positive rate of binding sites, we chose a relative strict cutoff score for each TFBS. At a cutoff score of 0.92, we estimated the fraction of false positive predictions to be less than 5% of the total number of planted sites on coding regions of human genes in the RefSeq database.

### DNA substitution models

PSPE is able to use many different commonly used models for nucleotide substitution, including Jukes-Cantor (JC) [66], Felsenstein 1981 (F81) [67], Kimura 2-parameter (K80) [68], Hasegawa-Kishino-Yano (HKY) [40], Tamura-Nei (TrN) [69], and the general time reversible (GTR) model [70-72]. The GTR model has eight free parameters with the following instantaneous substitution rate matrix:

$$Q=\left[\begin{array}{cccc} q_{aa} & \mu_{ac}\pi_{c} & \mu_{ag}\pi_{g} & \mu_{at}\pi_{t}\\ \mu_{ca}\pi_{a} & q_{cc} & \mu_{cg}\pi_{g} & \mu_{ct}\pi_{t}\\ \mu_{gg}\pi_{a} & \mu_{gc}\pi_{c} & q_{gg} & \mu_{gt}\pi_{t}\\ \mu_{tt}\pi_{a} & \mu_{tc}\pi_{c} & \mu_{tg}\pi_{t} & q_{tt} \end{array}\right]$$

where *μ*_*ij *_is the instantaneous substitution rate of nucleotide *i *by *j*, *π*_*i *_is the frequency of nucleotide *i*, and $q_{ii}=\sum_{j\neq i}\mu_{ij}\pi_{j}$ where *i *= *a*, *c*, *t*, *g*; *j *= *a*, *c*, *t*, *g*. The other substitution models above can be expressed as special cases of the GTR model. From the Q matrix, we can obtain the matrix of nucleotide transition probabilities in continuous time by:

*P*(*t*) = *e*^*kQrt*^

where *k *is a correction factor, which is used to scale the substitution matrix such that branch lengths represent the expected number of substitutions per site, and *r *is the relative substitution rate to model heterogeneous substitution rates among different sites. Based on the Γ+I model [73,74], the relative rates at each position follow the same independent and identical distribution as defined by:

$$f(r|\alpha ,\iota )=\{\begin{array}{ll} 0 & if\;r<0\\ \iota & if\;r=0\\ \left(1-\iota ){\left(\alpha \cdot r\right)}^{\alpha }e^{-\alpha r}/r\Gamma (\alpha \right) & if\;r>0 \end{array}$$

where ι is the proportion of invariant rates and α is the shape parameter of the gamma distribution.

### InDel models

PSPE is based on Dawg [75], an earlier sequence evolution simulation system, and in particular adopted its range of InDel formation model. The model is based on a Poisson process that assumes InDel formation to happen at a fixed, instantaneous rate at any site. The model treats insertions and deletions as two separate processes. Under the model, the time intervals between two insertions and those between two deletions follow exponential distributions with means [λ_Ins _(*L *+ 1)]^-1 ^and [λ_Del _(L + u - 1)]^-1^, respectively, where L is the sequence length, u is the mean length of deletion segments, and λ_Ins _and λ_Del _are Poisson rates of insertion and deletion, respectively.

PSPE models InDel length by one of three commonly used distributions: geometric, negative binomial, and Zipf's law distributions. We used the simple geometric distribution for InDel length in this study.

### Simulation of sequence evolution

To address TFBS turnover at different distances, we simulated sequence evolution at each of 15 different divergence distances: 0.01, 0.05, 0.1, 0.25, 0.5, 0.75, 1.0, 1.25, 1.5, 1.75, 2.0, 2.5, 3.0, 4.0, and 5.0, measured in the number of substitutions per site. These distances should cover the divergence between most currently sequenced genomes used in comparative genomics studies. To study the effect of different InDel rates on TFBS conservation between human and mouse, we performed simulations at ten different InDel rates measured by the number of InDels per substitution: 0, 0.01, 0.025, 0.05, 0.075, 0.1, 0.125, 0.15, 0.175, 0.2, with, for example, 0 meaning no InDel, and 0.2 meaning one InDel every five substitutions. For studying the effects of restricted translocation distances on the RTRs, we compared RTRs of each TFBS at 12 different maximal translocation distances: 0, 5, 10, 20, 30, 40, 50, 75, 100, 150, 200, and 300 bases.

### Model fitting method

To estimate parameters of our RTR models, we did a non-linear least-squares regression analysis on the observed turnover data from simulation. We used the Gauss-Newton algorithm for the non-linear least-squares fitting, which minimizes the sum-of-squares error. We performed this analysis in R.

### Alignment tools

We selected five alignment tools, CLUSTALW, DIALIGN, AVID/MAVID, LAGAN/MLAGAN, and MUSCLE, to assess their capability of detecting functional sites. The criteria for our selection were: either widely used or shown good performance in other studies for aligning DNA sequences; capable of aligning multiple sequences in a reasonable amount of time; free availability and easy installation in the Linux operating system; and strict co-linearity for global alignment. Many excellent alignment tools were not evaluated because: they did not meet one of the above criteria; their algorithms were similar to one of five tools we selected; and/or they did not show a significantly different performance in other evaluations. For example, ACANA [21], BLASTZ [76], MUMmer [77], and SSEARCH [78] were not selected because of the first criterion, and T-COFFEE [22], POA [79] and MAFFT [80] were not evaluated due to the second criterion. Our study is therefore not considered to be a systematic evaluation of all available good alignment tools and rather as a representative but somewhat subjective cross-section. Each of the five tools is briefly described below.

CLUSTALW (v1.83) is one of the best-known MSA tools and is based on a progressive method, which first aligns the most similar sequences and then successively adds more distant sequences or groups to the alignment until all sequences are aligned together. CLUSTALW employs the Needleman-Wunsch pair-wise alignment algorithm for calculating similarities between sequences and constructing a phylogenetic guide tree. We ran CLUSTALW with its default settings.

DIALIGN (v2.2.1) is an anchor-based MSA tool for both DNA and protein sequences. Different from other alignment tools, DIALIGN assembles alignments by finding consistent segments exhibiting statistically significant similarity, and does not align regions showing no significant similarity. Therefore, strictly speaking, DIALIGN is a local alignment tool that produces a full global alignment only for sequences with high similarity. Recent studies [21,28,79] suggested that DIALIGN performed well in aligning sequences of low similarity with long insertions and deletions. DIALIGN was run with default parameters.

AVID (v2.1) is a pair-wise global alignment tool that is capable of aligning very large genomics sequences. Its employs an anchor-based approach and uses a suffix tree algorithm for identifying potential anchoring regions between sequences. MAVID (v2.0.4) is a progressive MSA tool and is the direct extension of AVID. To speed up the alignment process, MAVID does not directly align two intermediate alignments or groups; instead, it first infers the common ancestral sequence of each alignment by maximum likelihood, and then uses AVID to align two ancestral sequences. We used AVID for two-species alignments, and MAVID for three or more species. CLUSTALW at default settings was used as the tree building tool for MAVID.

LAGAN/MLAGAN (v1.21) is a suite of programs for aligning DNA sequences. As an anchor-based pair-wise alignment tool, LAGAN first identifies anchoring regions by chaining similar neighboring words found in a local alignment process, and subsequently aligns other regions by the Needleman-Wunsch algorithm to form a global alignment. MLAGAN is a progressive multiple sequence alignment tool based on LAGAN pair-wise alignments. Similar to CLUSTALW in scoring multiple sequence alignments, it uses the sum-of-pairs approach for scoring substitutions and a consensus-based method for scoring gaps. Since MLAGAN does not build a phylogenetic tree, which is a required input, we provided it with the phylogenetic tree from our simulation. In this study, LAGAN was used for two-species alignments and MLAGAN for three or more species. Both tools were run with default settings.

MUSCLE (v3.6) is a relatively new MSA tool for both DNA and protein sequences based on an improved progressive approach. Like CLUSTALW and T-COFFEE, MUSCLE is based on a progressive approach, and uses the sum-of-pairs scoring scheme for multiple alignments, but it differs from other progressive tools by allowing changes of both the phylogenetic tree and the alignment in intermediate steps in an iterative refining process. MUSCLE was shown to perform better than T-COFFEE and POA in aligning benchmark protein sequences [20,81]. Because MUSCLE is quite slow when running on default settings, we used its *diags *option for anchoring alignment and *maxiters *option to limit the number of refinement iterations to two.

### Simulation of alignment benchmark data

The benchmark sequence datasets were simulated by the PSPE system. PSPE sequence simulation can be generally divided into two separate steps. In the first step, PSPE generates ancestral promoter sequences with different functional TFBSs (see Table 6 for the sites we used in this study). With the exception of YY1E2F, a composite of YY1 and E2F and chosen on purpose for this study, the TFBS sites used here were arbitrarily selected among those satisfying three criteria: binding site of a human transcription factor; PWM available in the JASPAR database; and length between 12 and 25 bp. A motif instance, which is generated randomly from a PWM, is defined as a functional site if its score is larger than a certain cutoff value. To avoid degenerate motifs, we used a relatively stringent cutoff of 0.90 for all TFBSs. PSPE first generates functional sites and a map of their locations, and then fills the remaining region in promoters with background sequences. To generate biologically relevant background sequences, we estimated parameter values of a 3rd order Markov chain from a large real sequence data set and used them to simulate the ancestral background sequences. The actual sequence data consisted of human promoter sequences of 24,649 transcripts extracted from the NCBI human RefSeq database (build 35). Each sequence in the training data comprises a 5,000 bp region upstream of the putative transcription start site.

In the second step, PSPE simulates descendent sequences from the simulated ancestral promoter sequences according to specified evolutionary models and functional constraints (Table 5). For the evaluation on the mammalian tree of five species, we scaled the tree, relative to evolutionary distance, at the following eight levels: 0.25, 0.5, 1, 1.5, 2, 3, 4, and 5, which we refer to as divergence scale coefficient. At each scale level, PSPE generated 1,000 sets, each consisting of five descendent mammalian sequences from their common ancestral sequence of length 3,000 bp.

### Performance measures

We use the expressions 'a tool alignment' to refer to an alignment produced by an alignment tool, 'a simulated alignment' to refer to a correct alignment of homologous base pairs from the simulation, and 'a simulated TFBS map' to refer to a correct alignment of TFBSs homologous on the functional level. Both 'a simulated alignment' and 'a simulated TFBS map' were generated by the PSPE simulator. The ability of an alignment tool to detect functional sites was assessed by TFBS detection accuracy. Tool performance was also assessed by four additional measures: overall alignment sensitivity, overall alignment specificity, TFBS sensitivity and TFBS specificity, which are similar to measures in [28]. Definitions of these measures are given below.

TFBS detection accuracy (*DA*) is defined as the proportion of functional sites correctly aligned with respect to a simulated TFBS map, averaged over all different pairs in a multiple sequence alignment, and can be calculated by:

$$DA=\frac{2}{n(n-1)L}\sum_{\text{i}=1}^{\text{n}(\text{n}-1)/2}\sum_{j=1}^{L}a_{ij}$$

where *n *is the number of sequences in the alignment, *n(n-1)/2 *is the number of different sequence pairs, and *L *is the total length of functional sites (for a *DA *of an individual functional site, *L *is the length of the site). *a*_*ij *_has a value of *1 *if the bases at the *j*^th ^position of functionally homologous binding sites in a sequence pair *i *are aligned to each other and 0 otherwise. Since all our simulated sequences contain the same set of TFBSs, *L *is the same for all alignments.

The overall alignment sensitivity is the fraction of correctly aligned bases not considering gaps, averaged over all different pairs in a tool alignment:

$$overall\_sensitivity=\frac{2}{n(n-1)}\sum_{\text{i}=1}^{\text{n}(\text{n}-1)/2}\frac{1}{k_{i}}\sum_{j=1}^{k_{i}}c_{ij}$$

where *k*_*i *_is the total number of positions for which bases are aligned to bases in the *i*^th ^pair-wise alignment from a simulated alignment. *c*_*ij *_has a value of 1 if the *j*^th ^position was aligned correctly in any given pair of sequences *i *in the tool alignment, and 0 otherwise.

Overall alignment specificity is the fraction of correctly aligned bases among those that are aligned to gaps in a simulated alignment, averaged over all different pairs in a tool alignment:

$$overall\_specificity=\frac{2}{n(n-1)}\sum_{\text{i}=1}^{\text{n}(\text{n}-1)/2}\frac{1}{g_{i}}\sum_{j=1}^{g_{i}}c_{ij}$$

where *g*_*i *_is the total number of positions, where bases in one sequence aligned to gaps in the other sequence in the *i*^th ^pair-wise alignment of a simulated alignment; *c*_*ij *_has a value of 1 if the *j*^th ^position aligned correctly, and 0 otherwise.

TFBS sensitivity is the fraction of correctly aligned bases, among those in functional sites of ancestral sequences and not aligned to a gap in a simulated alignment, averaged over all different pairs in a tool alignment:

$$tfbs\_sensitivity=\frac{2}{n(n-1)}\sum_{\text{i}=1}^{\text{n}(\text{n}-1)/2}\frac{1}{l_{i}}\sum_{j=1}^{l_{i}}c_{ij}$$

where *l*_*i *_is the total number of positions in functional sites of ancestral sequences, where bases in one sequence aligned to bases from the other sequence in the *i*^th ^pair-wise alignment of a simulated alignment; *c*_*ij *_has a value of 1 if the *j*^th ^position aligned correctly, and 0 otherwise. In case of no replacement turnovers of TFBSs, TFBS sensitivity is equal to TFBS detection accuracy.

TFBS specificity is the fraction of correctly aligned bases, among those in functional sites of ancestral sequences and aligned to gaps in a simulated alignment, averaged over all different pairs in a tool alignment:

$$tfbs\_specificity=\frac{2}{n(n-1)}\sum_{\text{i}=1}^{\text{n}(\text{n}-1)/2}\frac{1}{L-l_{i}}\sum_{j=1}^{L-l_{i}}c_{ij}$$

where *L *is total length of all functional sites in the ancestral sequence.

In this paper, we mostly use detection accuracy and TFBS sensitivity. The former refers to the fraction of functional sites in the 'descendants' aligned to each other, the latter to the fraction of sites corresponding to the location of the TFBS in the 'ancestral' sequence that are correctly aligned to each other.

## Abbreviations

GTR, general time reversible; HKY, Hasegawa-Kishino-Yano; InDel, insertion/deletion; MSA, multiple sequence alignment; PSPE, Phylogenetic Simulation of Promoter Evolution; PWM, position weight matrix; RTR, replacement turnover rate; TFBS, transcription factor binding site; TSS, transcription start site.

## Authors' contributions

WH, JN and UO contributed to the conception and design of this study. WH developed PSPE, performed analyses, and drafted the initial manuscript. WH and UO provided the interpretation of results. All authors contributed to writing and critically revising the manuscript. All authors read and approved the final manuscript.

## Additional data files

The following additional data are available with the online version of this paper. Additional data file 1 provides additional results of turnover simulations varying the binding site strength and GC content of the background sequences, and information on the E2F promoter data set. Additional data file 2 provides additional evaluations of alignment algorithms on sequence sets simulated with a phylogenetic tree with a star topology.

## Supplementary Material

Additional data file 1 Additional results of turnover simulations varying the binding site strength and GC content of the background sequences, and information on the E2F promoter data set. Click here for file

Additional data file 2 Additional evaluations of alignment algorithms on sequence sets simulated with a phylogenetic tree with a star topology. Click here for file
