# Supplementary material for: Phylogenetic simulation of promoter evolution: estimation and modeling of binding site turnover events and assessment of their impact on alignment tools
Source: Genome Biol. 2007 Oct 24;8(10):R225. doi: 10.1186/gb-2007-8-10-r225 (PMC2246299; doi:10.1186/gb-2007-8-10-r225)
Supplement: Additional data file 1 — Additional results of turnover simulations varying the binding site strength and GC content of the background sequences, and information on the E2F promoter data set. [file gb-2007-8-10-r225-S1.pdf]

Supplementary material for TFBS replacement turnover rate study

Weichun Huang[1], Joseph R Nevins, and Uwe Ohler
Institute for Genome Sciences and Policy, Duke University, Durham, NC 27708
[1]Current address: Department of Biology, Boston College, Chestnut Hill, MA 02467

List of human cell-cycle genes under transcriptional regulation by E2F

Table 1: List of human cell-cycle genes under transcriptional regulation by E2F used for evolution simulation. All genes in the list have one and only one E2F binding site in upstream 500 *bps* region when using a cutoff score of 0.92. These genes were used to estimate the replacement turnover rates starting from real ancestral promoters.

Refseq Acc #	Gene Symbol	E2F Location	E2F Score	Gene name
NM_001254	CDC6	-8	1.00	CDC6 cell division cycle 6 homolog
NM_000321	RB1	-49	0.93	retinoblastoma protein 1
NM_002689	POLA2	-52	0.98	polymerase (DNA directed), alpha 2 (70kD subunit)
NM_003362	UNG	-93	0.94	uracil-DNA glycosylase (UNG)
NM_002388	MCM3	-132	0.93	MCM3 minichromosome maintenance deficient 3
NM_001789	CDC25A	-150	1.00	cell division cycle 25A (CDC25A)
NM_003071	SMARCA3	-197	0.93	SWI/SNF related, matrix associated, actin dependent regulator of chromatin, subfamily a, member 3
NM_003188	MAP3K7	-201	0.93	mitogen-activated protein kinase kinase kinase 7
NM_021238	FAM60A	-219	0.93	family with sequence similarity 60, member A
NM_001809	CENPA	-229	0.93	centromere protein A (CENPA)
NM_005956	MTHFD1	-286	0.93	methylenetetrahydrofolate dehydrogenase 1

Comparison of TFBS turnover rates from two simulations with different GC contents

Table II: Comparison of E2F replacement turnover rates from two simulations with different GC contents. The table shows that replacement turnover rate of E2F sites is generally lower in promoters with higher GC content. The SD is the standard deviation of mean.

Divergence Distance	Replacement Turnover Rate					
	GC=0.572			GC=0.484		
	median	mean	SD	median	mean	SD
0.01	0.000	0.0009	0.0020	0.000	0.0009	0.0020
0.05	0.003	0.0062	0.0091	0.003	0.0062	0.0095
0.10	0.006	0.0106	0.0136	0.006	0.0116	0.0161
0.25	0.016	0.0261	0.0283	0.018	0.0280	0.0288
0.50	0.033	0.0499	0.0429	0.038	0.0523	0.0395
0.75	0.051	0.0689	0.0493	0.055	0.0740	0.0491
1.00	0.071	0.0897	0.0526	0.075	0.0957	0.0534
1.25	0.086	0.1064	0.0548	0.094	0.1157	0.0575
1.50	0.103	0.1270	0.0613	0.115	0.1372	0.0591
1.75	0.120	0.1425	0.0636	0.130	0.1568	0.0641
2.00	0.132	0.1573	0.0646	0.148	0.1751	0.0669
2.50	0.166	0.1921	0.0693	0.183	0.2079	0.0675
3.00	0.195	0.2181	0.0671	0.218	0.2428	0.0681
4.00	0.253	0.2773	0.0677	0.277	0.2998	0.0662
5.00	0.304	0.3262	0.0677	0.334	0.3575	0.0650

Table III: Comparison of Myc replacement turnover rates from two simulations with different GC contents. The table shows that replacement turnover rate of Myc sites is slightly lower in promoters with higher GC content. The SD is the standard deviation of mean.

Divergence Distance	Replacement Turnover Rate					
	GC=0.572			GC=0.484		
	median	mean	SD	median	mean	SD
0.01	0.002	0.0025	0.0017	0.002	0.0025	0.0017
0.05	0.012	0.0125	0.0045	0.012	0.0123	0.0044
0.10	0.025	0.0247	0.0076	0.025	0.0248	0.0076
0.25	0.062	0.0590	0.0150	0.063	0.0599	0.0144
0.50	0.119	0.1135	0.0237	0.120	0.1140	0.0232
0.75	0.172	0.1630	0.0302	0.173	0.1637	0.0302
1.00	0.219	0.2094	0.0340	0.221	0.2105	0.0364
1.25	0.262	0.2499	0.0408	0.265	0.2536	0.0400
1.50	0.299	0.2879	0.0432	0.306	0.2935	0.0419
1.75	0.343	0.3297	0.0455	0.344	0.3302	0.0457
2.00	0.373	0.3601	0.0493	0.378	0.3644	0.0484
2.50	0.439	0.4253	0.0508	0.442	0.4274	0.0493
3.00	0.495	0.4816	0.0495	0.503	0.4881	0.0500
4.00	0.580	0.5691	0.0504	0.594	0.5818	0.0481
5.00	0.660	0.6489	0.0458	0.667	0.6554	0.0446

Table IV: Comparison of NFκB replacement turnover rates from two simulation with different GC contents. The table shows that replacement turnover rate of NFκB sites, different from E2F and Myc, is generally higher in promoters with higher GC content. The SD is the standard deviation of mean.

Divergence Distance	Replacement Turnover Rate					
	GC=0.572			GC=0.484		
	median	mean	SD	median	mean	SD
0.01	0.000	0.0010	0.0013	0.000	0.0009	0.0013
0.05	0.004	0.0059	0.0054	0.003	0.0054	0.0052
0.10	0.008	0.0117	0.0098	0.007	0.0107	0.0098
0.25	0.022	0.0298	0.0216	0.018	0.0257	0.0203
0.50	0.042	0.0573	0.0366	0.034	0.0487	0.0334
0.75	0.062	0.0809	0.0448	0.053	0.0701	0.0423
1.00	0.083	0.1023	0.0517	0.072	0.0919	0.0514
1.25	0.105	0.1278	0.0581	0.091	0.1122	0.0568
1.50	0.129	0.1514	0.0635	0.106	0.1307	0.0613
1.75	0.154	0.1737	0.0655	0.124	0.1502	0.0676
2.00	0.167	0.1915	0.0686	0.142	0.1710	0.0713
2.50	0.206	0.2323	0.0768	0.172	0.2025	0.0776
3.00	0.240	0.2658	0.0793	0.197	0.2323	0.0799
4.00	0.308	0.3299	0.0798	0.257	0.2882	0.0816
5.00	0.370	0.3910	0.0796	0.316	0.3480	0.0831

Comparison study of TFBS replacement turnover rates using different cutoff thresholds for functional sites

For the study of replacement turnover rates (RTR) in the main manuscript, we used stringent cutoff thresholds for three TFBS to ensure we have a low rate of false positive sites (see Figure 3 in the main manuscript). To study how TFBS RTR can be affected by its cutoff threshold or, in another term, its degeneracy, we performed an additional simulation, using an arbitrarily lower cutoff threshold 0.85 for all three binding sites. The results are summarized in the following three figures, in which the upper panel shows the simulation results using the higher cutoff threshold from the paper and the lower panel the results for the lower cutoff threshold. We found that RTRs of both E2F and NFκB sites increased with the lower cutoff threshold, while the RTR of Myc sites decreased slightly. The increase of RTR with a lower cutoff threshold can be explained by the increased rate of new functional sites generated. However, the RTR reduction in the case of Myc was somewhat unexpected at first: Myc has more degenerate binding sites than E2F and NFκB, and thus a higher rate of generating new sites given the same cutoff threshold. Therefore, when the rate of generating new functional sites became very high, it appeared to have the effect of lowering the observed RTR. The main reason for this can likely be found in our particular simulation scenario, which included a strict restriction: a promoter was allowed to have exactly one functional binding site of the same transcription factor. When a descendent sequence generated more than one functional site in evolution simulation, the sequence was simply discarded and a new one simulated until the condition was satisfied. In the process, we therefore eliminated sequences with many turnover events and counted only the remaining ones satisfying the conditions for the RTR calculation. This explains why we had a lower RTR for Myc site when using a lower cutoff threshold. This is biologically meaningful if one thinks of our set of restrictions as implicitly specifying a fitness function. The high turnover rate of an important TFBS is then lethal to an organism in our setting, and the observed turnover events in extant genomes may not reflect the actual probability of such an event to occur.

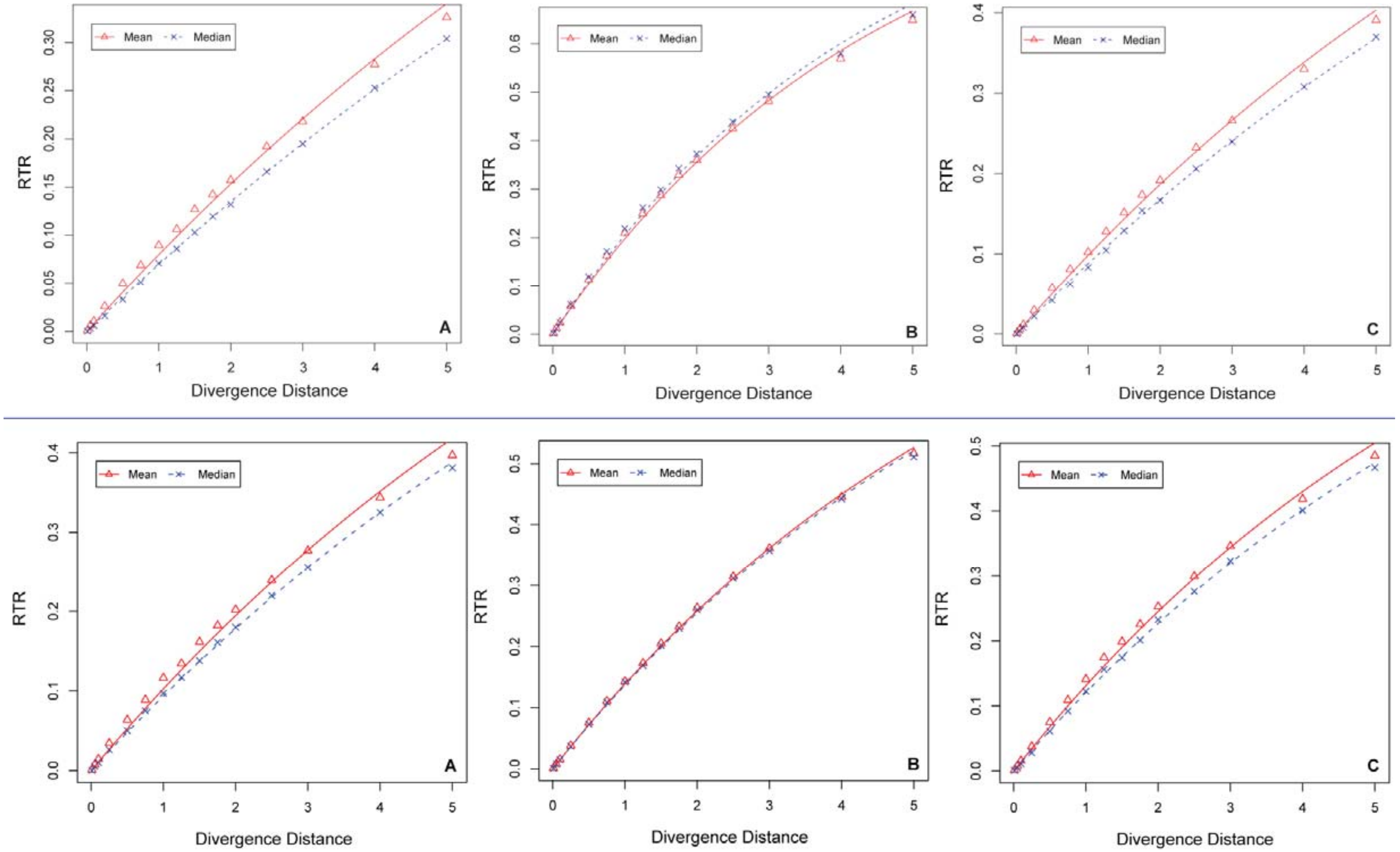

Figure 1: Relationship between evolutionary divergence distance and the replacement turnover rate (RTR) of transcription factor binding sites (TFBS). The upper panel shows the simulation results using the higher cutoff threshold of 0.92, and the low panel for the lower cutoff threshold of 0.85. The x-axis is the evolutionary divergence measured by the number of substitutions per site, and the y-axis is the RTR of a binding site. The points in the figure are observed RTRs from simulation, and lines are predicted RTR by our exponential models (equation 2) given in the main manuscript. The three TFBS are (A) E2F, (B) Myc and (C) NFκB.

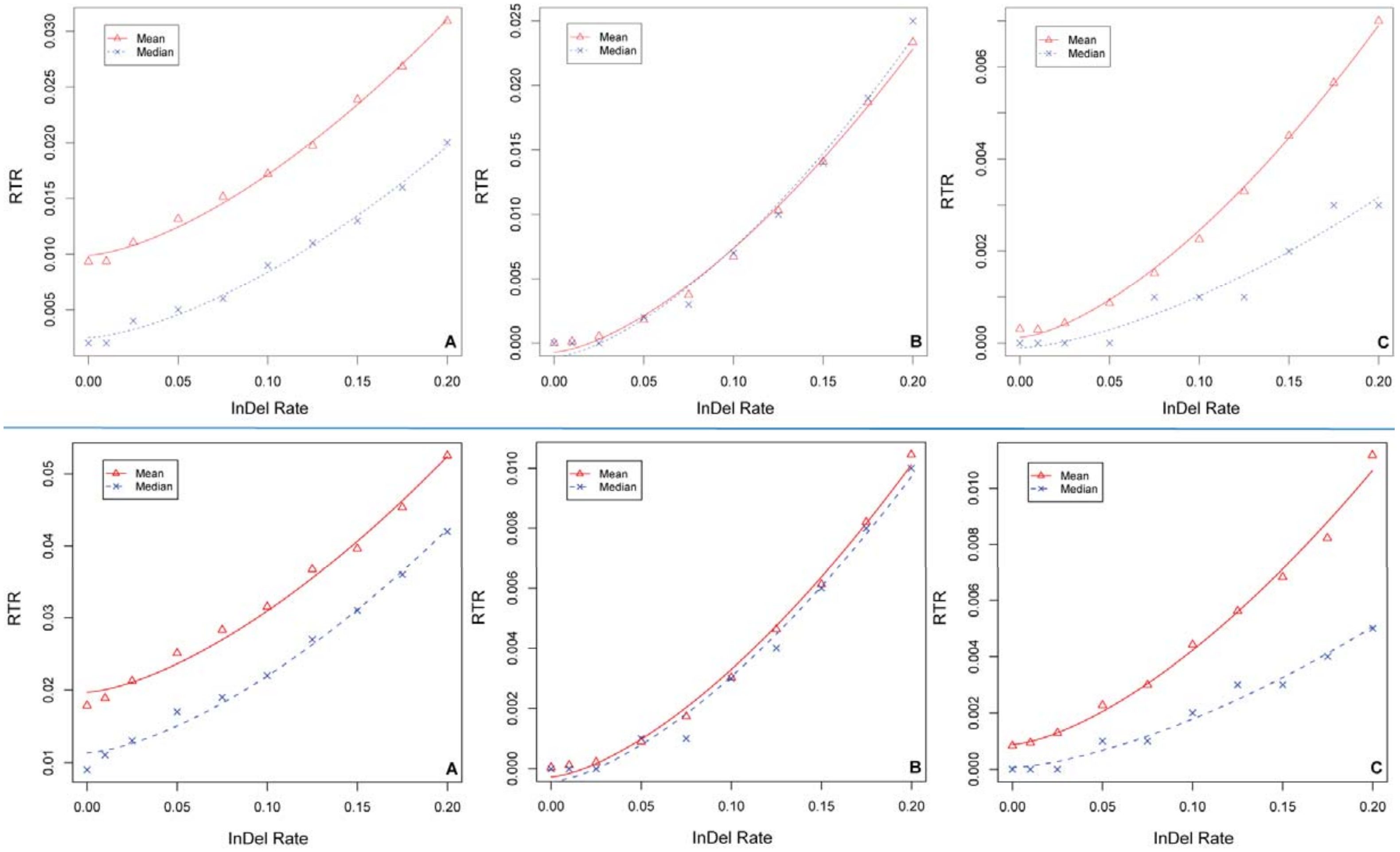

Figure 2: The effect of the Insertion/Deletion (InDel) rate on the RTR of TFBS. The upper panel shows the simulation results using the higher cutoff threshold and the low panel for the lower cutoff threshold. The x-axis is the InDel rate measured by the number of InDel events per substitution event, and the y-axis is RTR of TFBS. The points in the figure are observed RTRs from simulation, and lines show the RTR predicted by our model (equation 5) given in the main manuscript. The three transcription factor binding sites are (A) E2F, (B) Myc and (C) NFκB.

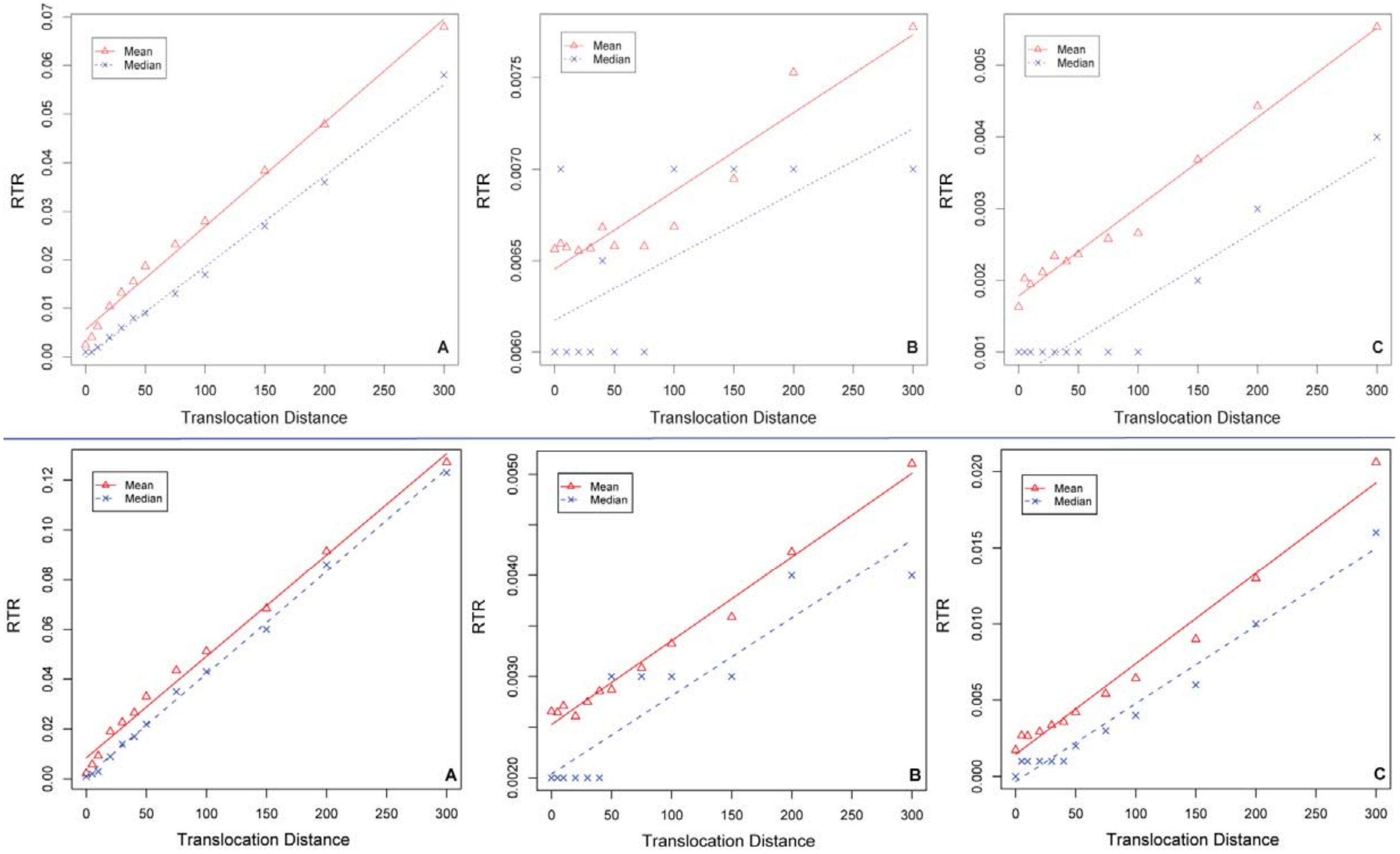

Figure 3: The effect of restricted translocation distance on the RTR of TFBS. The upper panel shows the simulation results using the higher cutoff threshold and the low panel for the lower cutoff threshold. The x-axis is the restricted translocation distance relative to the original binding site in the ancestral sequence, and the y-axis is the RTR of TFBS. The points in the figure are observed RTRs from simulation, and lines are predicted by our translocation RTR model (equation 6) given in the main manuscript. The three transcription factor binding sites are (A) E2F, (B) Myc and (C) NFκB.
