# Supplementary material for: Phylogenetic simulation of promoter evolution: estimation and modeling of binding site turnover events and assessment of their impact on alignment tools
Source: Genome Biol. 2007 Oct 24;8(10):R225. doi: 10.1186/gb-2007-8-10-r225 (PMC2246299; doi:10.1186/gb-2007-8-10-r225)
Supplement: Additional data file 2 — Additional evaluations of alignment algorithms on sequence sets simulated with a phylogenetic tree with a star topology. [file gb-2007-8-10-r225-S2.pdf]

Supplementary material for Alignment Tool Evaluation

Weichun Huang[1*], Joseph R Nevins, and Uwe Ohler[*]

Institute for Genome Sciences and Policy, Duke University, Durham, NC 27708
[1]Current address: Department of Biology, Boston College, Chestnut Hill, MA 02467

Evaluation on star tree simulation data

In addition to a mammalian tree topology, we used PSPE to generate benchmark promoter sequences based on a star phylogenetic tree at 15 different divergence distances (Supplementary Figure 1). The data were simulated under the HKY85 nucleotide substitution model with Gamma and invariant rate (Γ+I) for modeling substitution rate heterogeneity. For each divergence distance, we generated 1,000 replicate homologous sets, each having four promoter sequences with the same divergence distance from their ancestral sequence. Each sequence contained exactly one functional binding site for each of the six transcription factors: Pax6, TP53, IRF2, PPARG, ROAZ, and YY1E2F. YY1E2F is a composite TFBS consisting of YY1 and E2F binding sites that reportedly interact with each other in cell cycle gene regulation [48]. Binding sites were subject to a set of functional constraints (Supplementary Table 1) which were set to allow for turnover within a restricted distance, but keeping the overall order of the binding sites unchanged. Simulation allowed us to quantify the amount of turnover, how many non-aligned functional sites were due to turnover as compared to "simple" misalignments, and whether some tools would in fact be able to align functional sites despite turnover. We used this dataset to assess performances of five widely-used MSA tools: CLUSTALW, DIALIGN, AVID/MAVID, LAGAN/MLAGAN, and MUSCLE. The performance was measured as TFBS detection accuracy, defined as the proportion of nucleotides in functionally homologous TFBS which were correctly aligned. The detection accuracy reported here is the average value over 1,000 replicates at each divergence distance.

We compared the performance of the five tools in aligning sequences of two, three and four species, respectively. For two species (Supplementary Figure 2A), MUSCLE showed the highest overall detection accuracy (average over all of functional TFBS)

across different divergence distances; LAGAN/MLAGAN performed better than AVID/MAVID, CLUSTALW, and DIALIGN for sequences of intermediate and large divergence distances; and CLUSTALW was slightly better than DIALIGN and AVID/MAVID for sequences of short and intermediate divergence distances. For three (Supplementary Figure 2B) and four species (Supplementary Figure 2C) alignments, MUSCLE still had the best overall performance, but DIALIGN gradually overtook the other three tools, whose relative performance order with respect to each other remained unchanged. The TFBS detection accuracy decreased as divergence distances increased for all tools.

For each tool, there were also significant differences in performance on different TFBS, and differences became more pronounced as sequence divergence increased. For example, in four species alignment, all tools were better at aligning YY1E2F and Pax6, which had low replacement turnover rates and short restricted distance for translocation, than for IRF2 and ROAZ, which had higher turnover rates and long restricted distances for translocation (Supplementary Figure 3). Besides the restricted distance for translocation, other properties of TFBS, such as length, nucleotide composition and distance to neighboring TFBS, could have significant impact on its detection accuracy. For example, PPARG had a similar low turnover rate as TP53, but each tool had higher detection accuracy on TP53 than on PPARG. The degree of performance variation among TFBS was not always consistent among different tools; for instance, DIALIGN performed better on PPARG than MUSCLE, which had the highest detection accuracies for all other TFBS.

We also assessed the performance of each tool separately on aligning sequences of two, three and four species, respectively (Supplementary Figure 4). Contrary to the belief that more distantly related species help to locate functional conserved sites, we found that the increase in number of species did not necessarily increase the TFBS detection accuracies of all tools. AVID/MAVID and LAGAN/MLAGAN showed a decrease in performance as the number of species increased, and the decrease was more significant with increasing divergence distance. CLUSTALW showed the same tendency, but difference in

performance was less significant. Interestingly, MUSCLE had no significant difference in performance as the number of species increased, while DIALIGN improved its performance markedly across different divergence distances.

We made additional evaluations on three more promoter sequence datasets. These datasets were simulated using the same parameters as above except for one change each: the first one uses a zero order Markov model for background sequence simulation, the second does not use Γ+I for rate heterogeneity, and the third uses a different set of TFBS. The results were largely consistent with those reported above. Finally, we compared performances of the five tools in terms of their overall alignment sensitivity and TFBS sensitivity. We found that MUSCLE and CLUSTALW had slightly better overall alignment sensitivity than the other three, and the rank of TFBS sensitivities were in the same order as the detection accuracies (see Supplementary Figure 5).

Evaluation on mammalian tree simulation data

In addition to the star tree performance, we also report here additional results on data simulated using a mammalian tree. These data (Supplementary Figures 6-9) correspond to Figures 10-13 in the manuscript, but we scaled the mammalian tree at 2 additional levels and included the results at the scale factors of 7.5 and 10, relative to the distances shown in the tree (Supplementary Figure 1). Like this, we can observe the tool behavior on sequences in which anything but the functional sites is likely to have completely diverged.

Comparison of Tool Detection Accuracy of Turnover Sites only (mammalian tree simulation)

Supplementary Figures 10-16 complement Figures 10 and 11 in the paper, and show the performance when counting only turnover sites as positives. The evaluation is performed on the complete set of sequences, but different from the figures in the main paper, simuALN here refers to the subset of TFBS which arose from turnovers and trace back to the same ancestral site. As these sites share the same common ancestral nucleotides, they should be aligned to each other even though the site resides in a new location compared

to the site in the ancestor sequence we started from. More sites arise from turnovers as the divergence distance increases, and thus a larger fraction of sites fall into that category. Alignment tools can exceed the performance of simuALN if they align turnover nucleotides which do not trace back to the same position in the common ancestor.

Tables

Table 1: Functional TFBS constraints used in the promoter simulation. The accession numbers in the second column are from the JASPAR database. “Location” refers to the restriction on the upstream minimum and maximum distances to transcription start site. YY1E2F is a composite TFBS created by joining the YY1 and E2F sites.

Name	Accession #	Len	Strand	Location (min, max)	Copy # (min, max)	Cutoff
YY1E2F	MA0095 (YY1)<br>MA0024 (E2F)	13	+	(20, 30)	(1, 1)	0.90
Pax6	MA0069	14	+	(50, 70)	(1, 1)	0.90
TP53	MA0106	20	+	(360, 400)	(1, 1)	0.90
IRF2	MA0051	18	+	(420, 480)	(1, 1)	0.90
PPARG	MA0066	20	+	(2000, 2080)	(1, 1)	0.90
ROAZ	MA0116	15	+	(2100, 2200)	(1, 1)	0.90

Table 2: Simulation parameters used by PSPE for generating benchmark promoter sequences.

Evolution distance per step	0.05 substitution per site
Length of root sequences	3000 *bps*
Background sequence model	Markov Order of Third
Base frequencies	A=0.258, C=0.242, G=0.242, T=0.258
Substitution Model	HKY85
Transition/Transversion Ratio	20:1
Rate heterogeneity	Gamma (1.0) + Iota (0.1)
Range of GC content	(0.45, 0.55)
Gap model	Negative Binomial Distribution (1, 0.5)

Figures

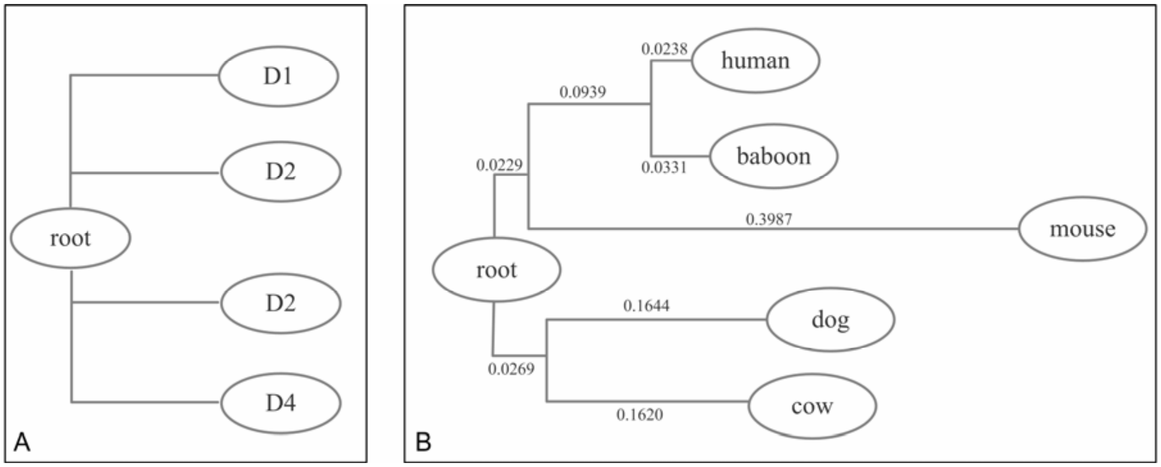

Figure 1: The two phylogenetic trees for promoter sequence simulation. (A) The star topology. In the star tree, four descendants (node D1 to D4) are evolved independently from the root sequence, and they have the same divergence distance from the root. We used D1 and D2 for two species alignments, and D1, D2 and D3 for three species alignment. (B) The phylogenetic tree of five mammals. The evolutionary distances shown in the tree were recently inferred from the coding region of orthologous genes [46]. In our simulation, we used the tree scaled at 10 different levels relative to the evolutionary distances shown.

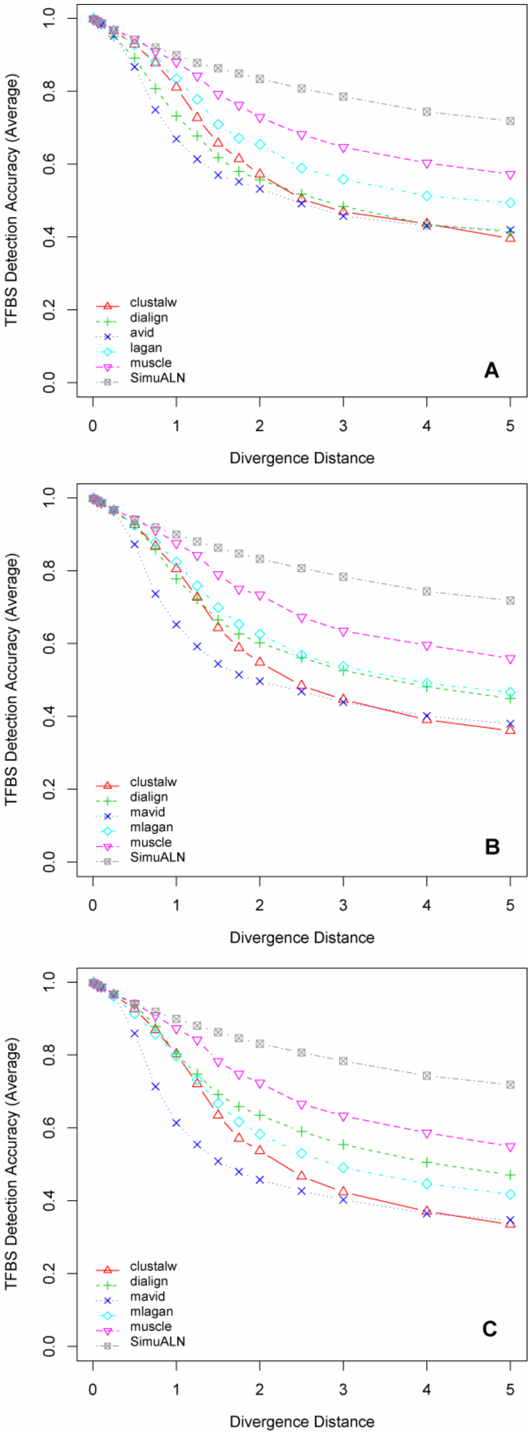

Figure 2: Performance comparison of alignment tools for TFBS detection accuracy. The Y-axis is the TFBS detection accuracy, the X-axis is the divergence distance measured by the number of substitutions per site. The SimuALN stands for the simulated alignment and its measure indicates the proportion of TFBS not subject to replacement turnover in the descendent sequences, and thus aligned in simulated alignments. (A) two species alignments, (B) three species alignments, and (C) four species alignments.

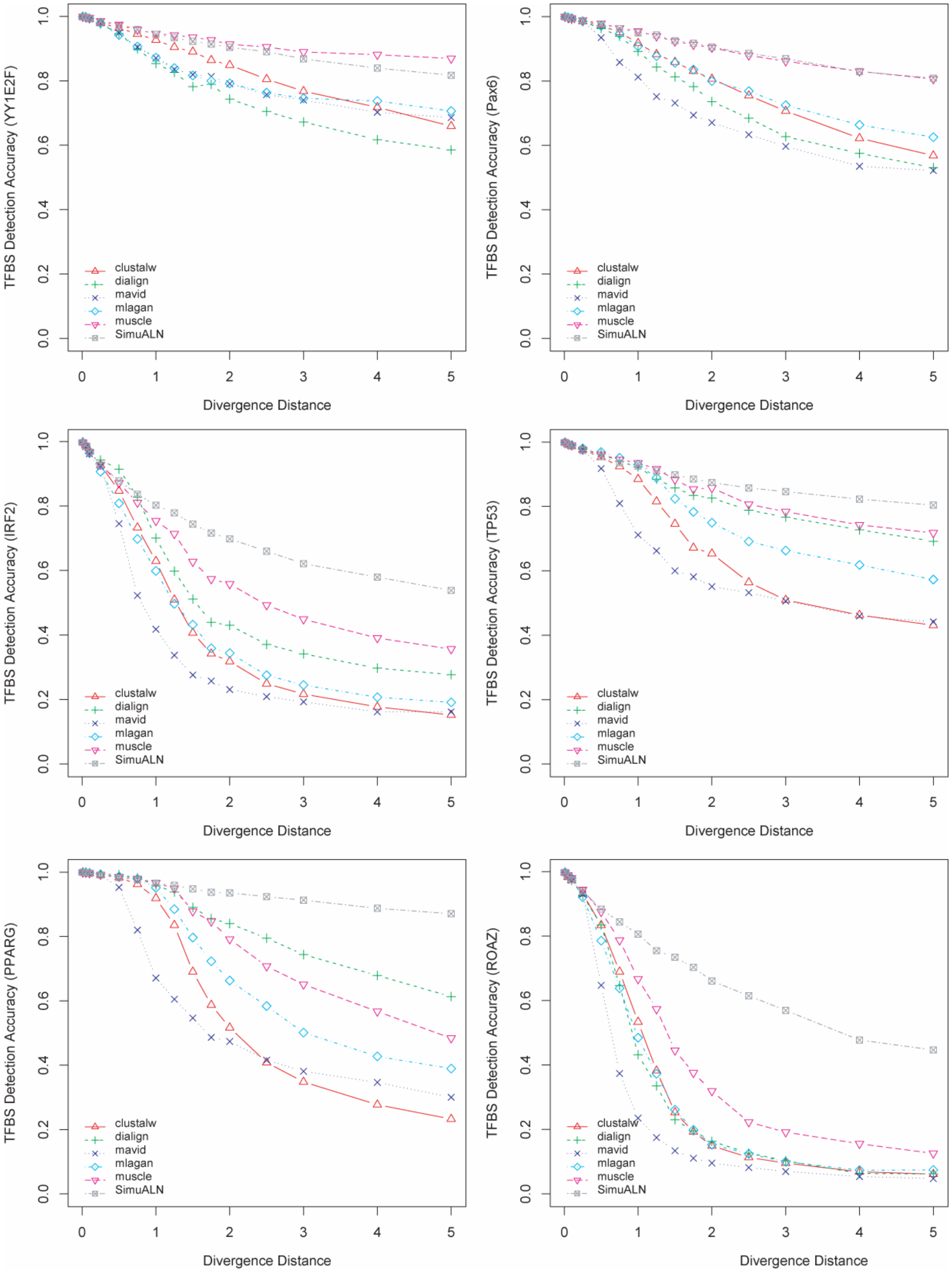

Figure 3: The detection accuracy on individual TFBS in four species alignments. All five tools perform better on detecting TFBS YY1E2F and Pax6, which have low replacement turnover rates and a short restricted distance for translocation, than on detecting IRF2 and ROAZ, which have high turnover rates and long restricted distances for translocation. Overall, MUSCLE performs superior to other four tools, while DIALIGN shows good performance on detecting TP53 and PPARG, which have long restricted translocation distances but relatively low replacement turnover rates.

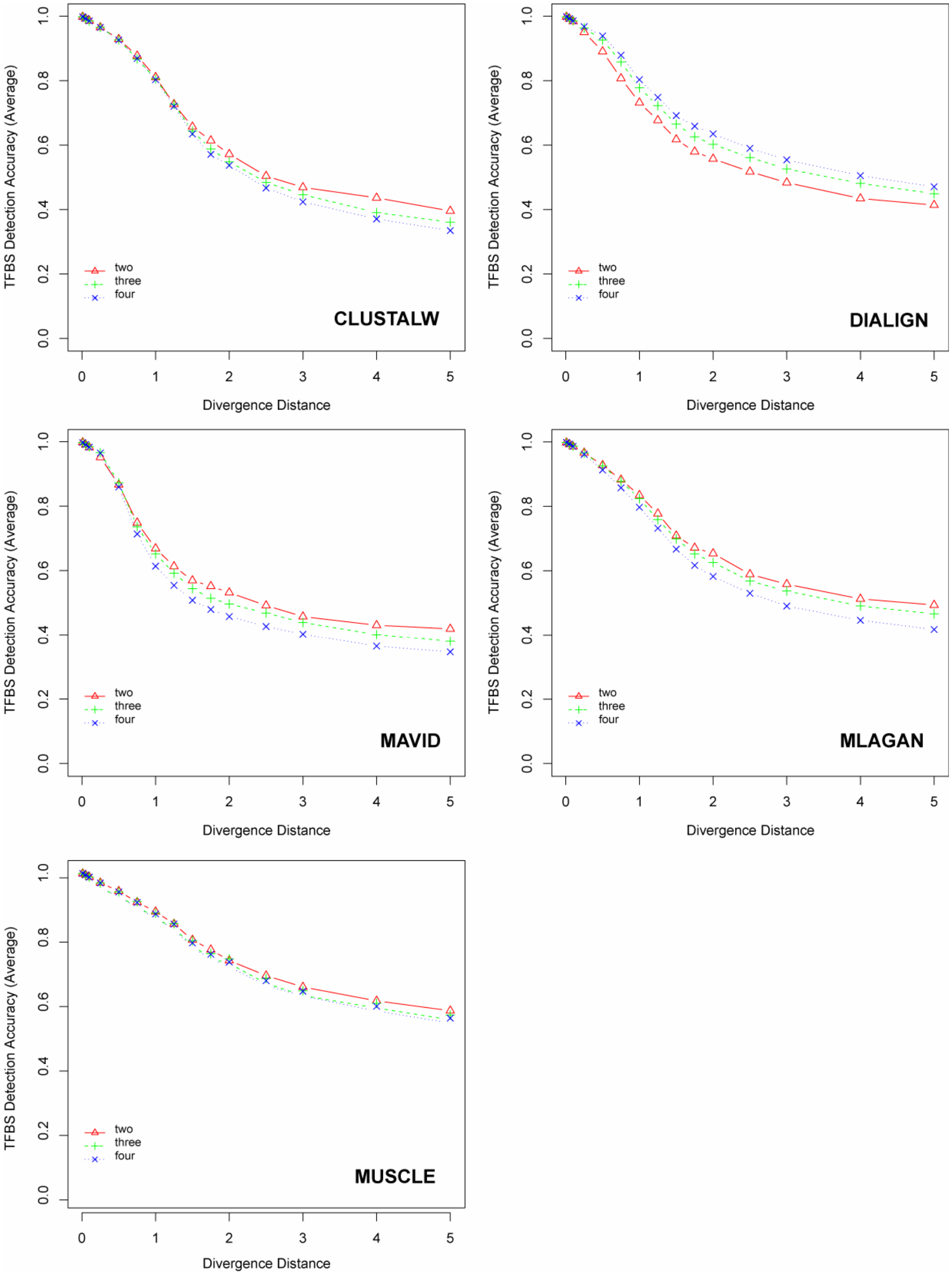

Figure 4: The effects on TFBS detection accuracy of five alignment tools as the number of species increases. Each subfigure shows a comparison of TFBS detection accuracy of the tools on aligning promoter sequences of two, three, and four species, respectively. The figure shows that the performances of CLUSTALW, AVID/MAVID and LAGAN/MLAGAN decrease as the number of species increases, especially at large divergence distances. The performance of MUSCLE is relatively unaffected; only DIALIGN shows improvement.

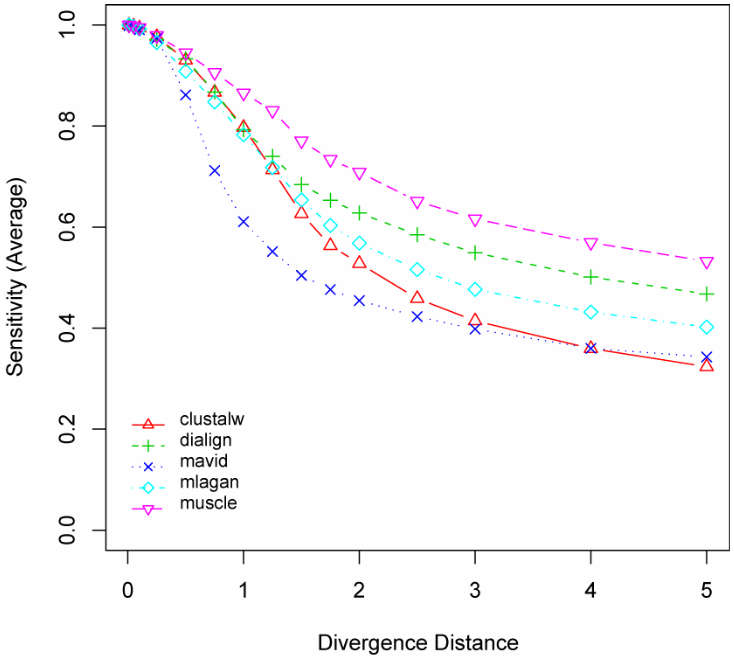

Figure 5: The average alignment sensitivity of TFBS on four species alignment. The relative order of TFBS sensitivity is almost identical to the order of TFBS detection accuracy (see Figure 2C).

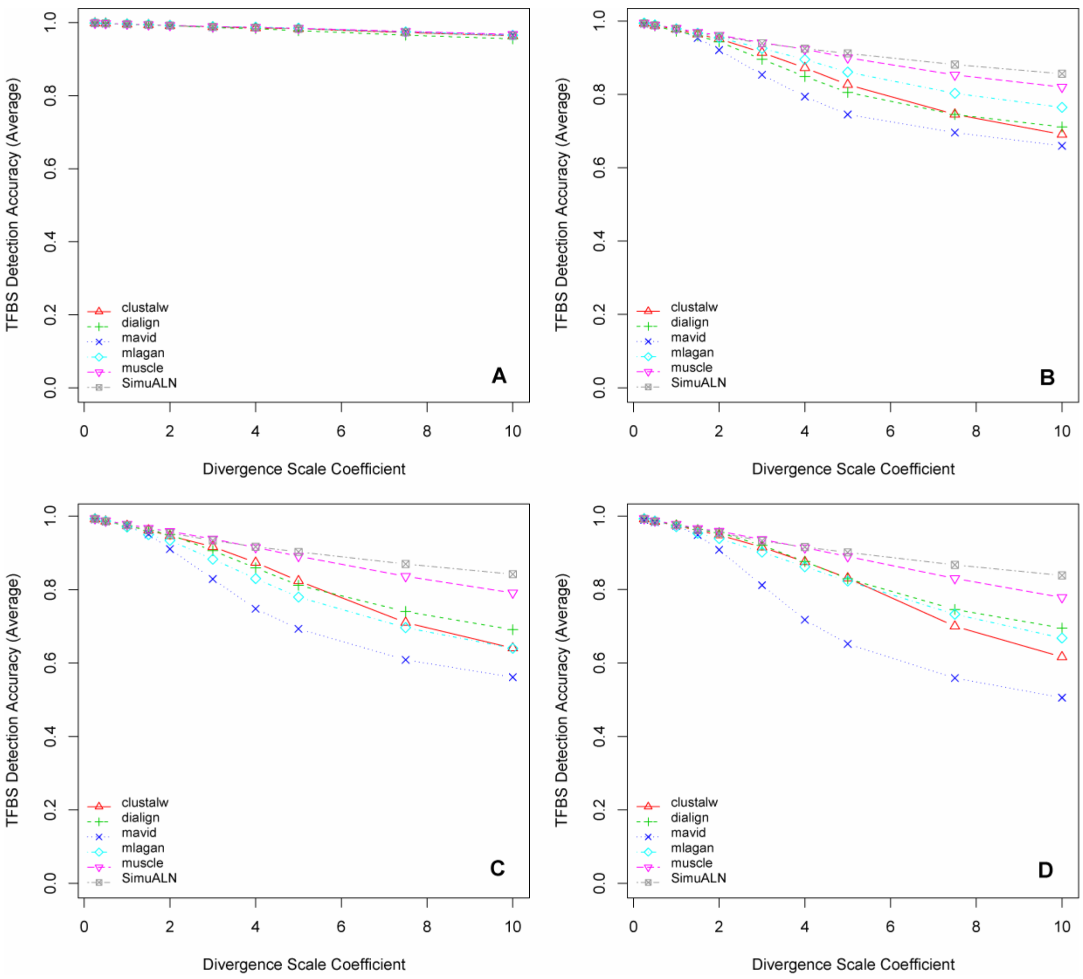

Figure 6: The average TFBS detection accuracy of five tools for mammalian sequence alignment. The Y-axis is the TFBS detection accuracy average on six TFBS, and the X-axis is the divergence scale coefficient of the mammalian phylogenetic tree (Figure 2B). The SimuALN stands for the simulated alignment and its measure indicates the proportion of TFBS not subject to replacement turnover in descendent sequences, and thus aligned in simulated alignments. (A) Two species alignments of human and baboon. (B) Three species alignments of human, baboon and mouse. (C) Four species alignments of human, baboon, mouse, and dog. (D) Five species alignment.

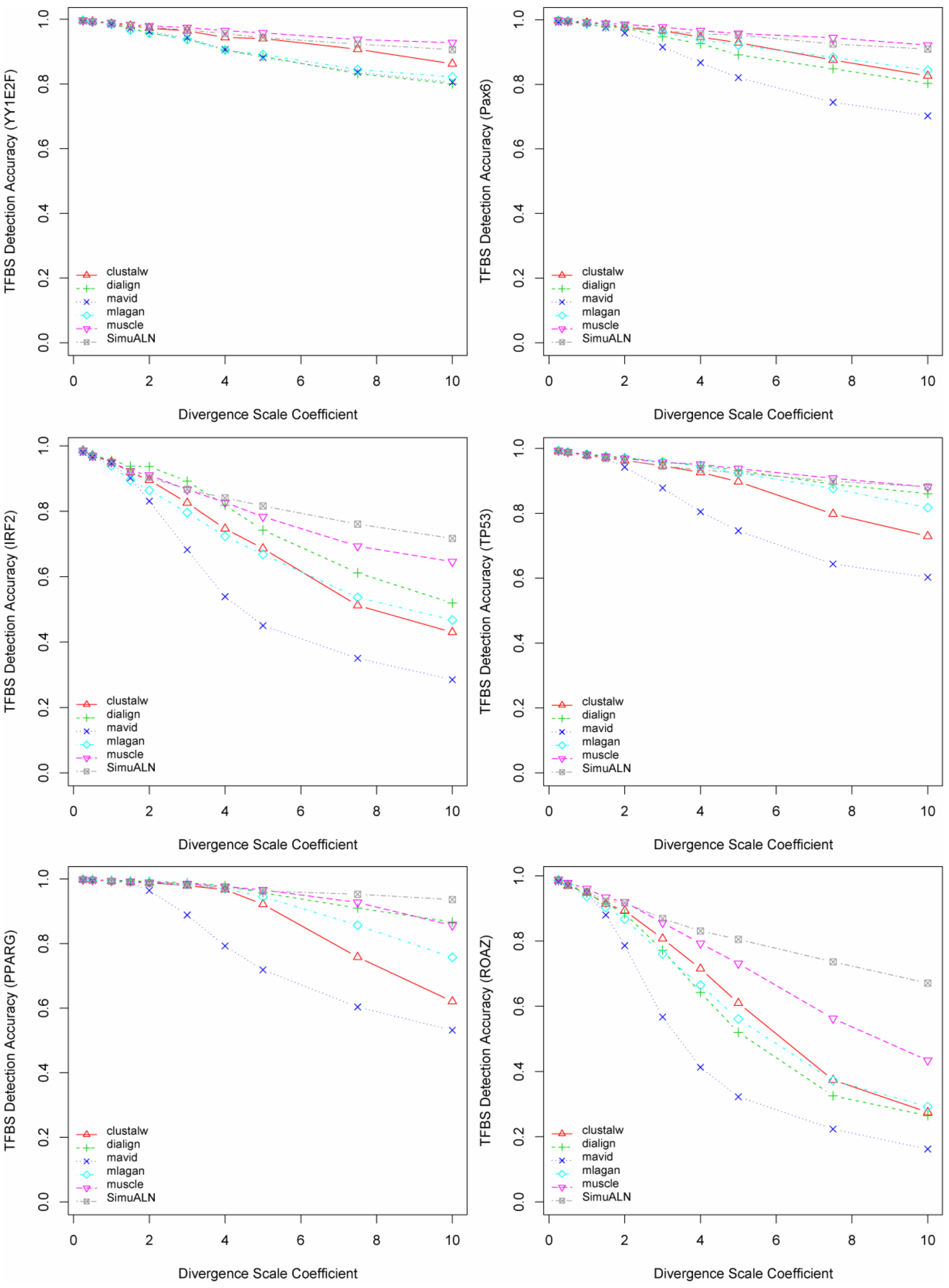

Figure 7: The detection accuracy of individual TFBS on five-way mammalian alignments. All five tools perform better on detecting YY1E2F and Pax6 which have low replacement turnover rates and short restricted distance for translocation than on detecting IRF2 and ROAZ which have high turnover rate and long restricted distance for translocation. MUSCLE shows an overall better performance than the other four tools. MLAGAN performs better than DIALIGN on YY1E2F, PAX6, PPARG and ROZA, while DIALIGN shows a better performance than MLAGAN on TP53 and PPARG, which have a long restricted distance for translocation but a relatively low replacement turnover rate.

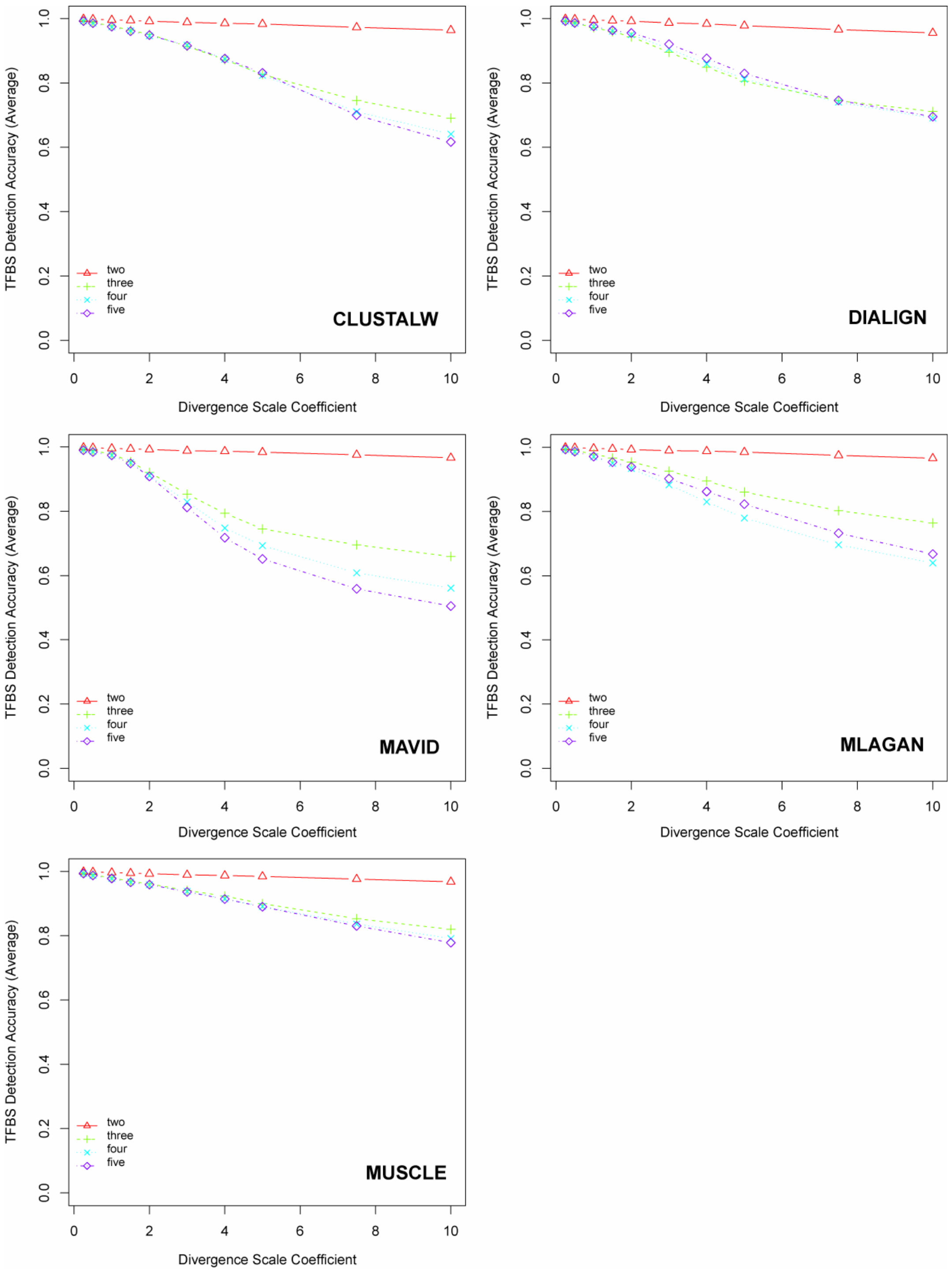

Figure 8: The effects of the number of aligned mammalian species on the TFBS detection accuracy. Each subfigure shows the performance of a tool in aligning a different number of species. Human and baboon were used for two species alignment, mouse was added for three species alignment, all five species but cow were used for four species alignment. While all tools have almost the same performance for aligning the two closely related species human and mouse, MUSCLE and DIALIGN perform better than other tools in maintaining or improving performance when adding more species to the alignment.

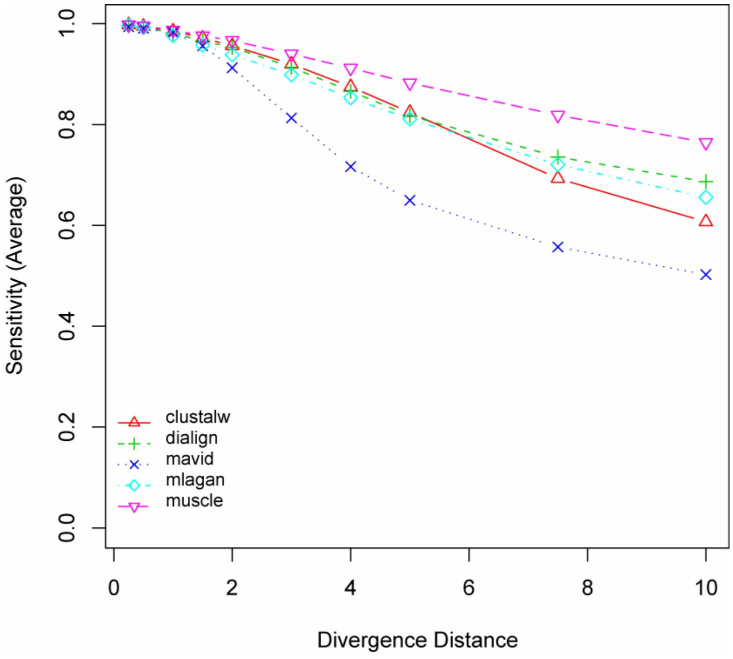

Figure 9: The average TFBS sensitivity of five tools on aligning TFBS in five mammalian species. The relative order on TFBS sensitivity of five tools is almost the same as the order on their TFBS detection accuracy (see Figure 6D).

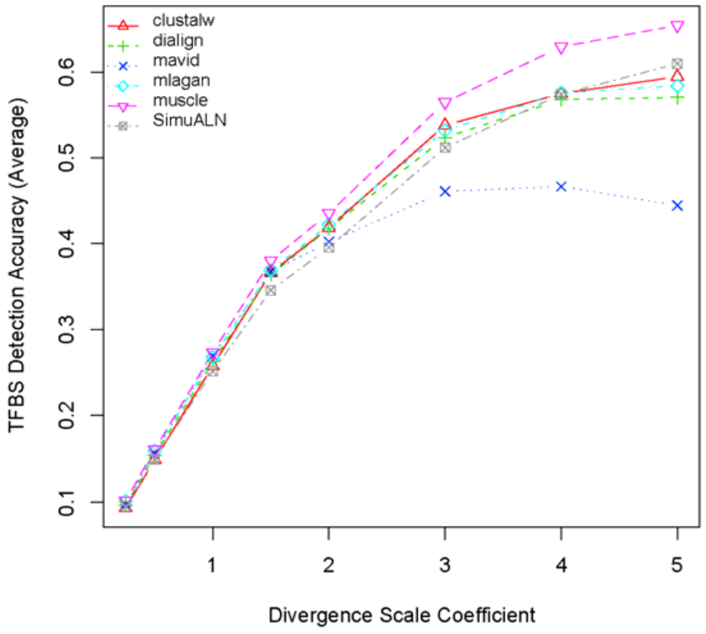

Figure 10: Comparison of the detection accuracy of all turnover sites

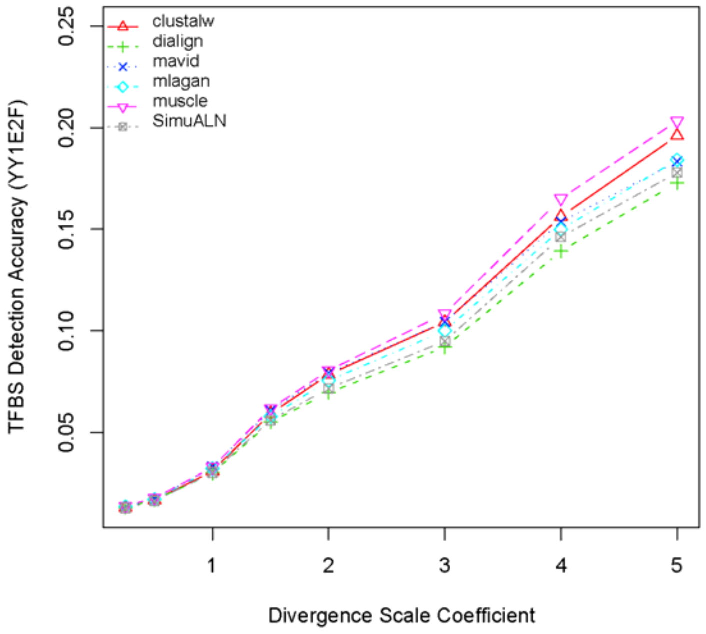

Figure 11: Comparison of the detection accuracy of YY1E2F turnover sites

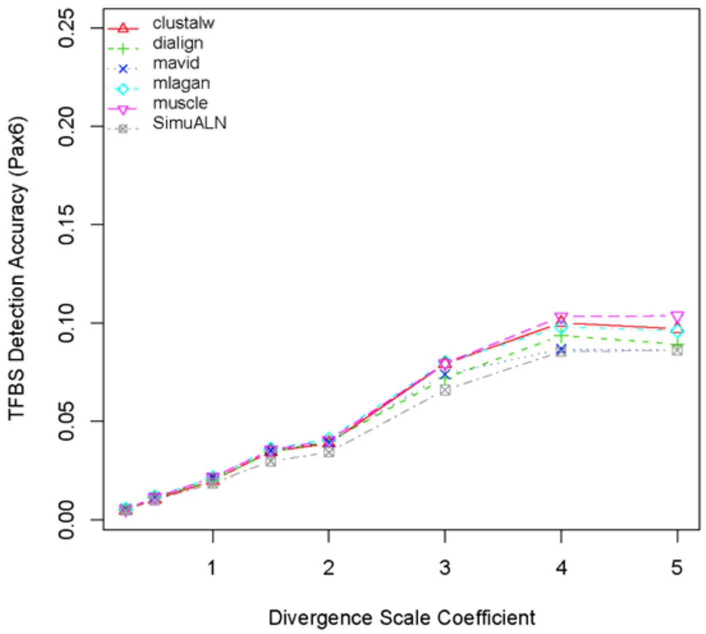

Figure 12: Comparison of the detection accuracy of Pax6 turnover sites

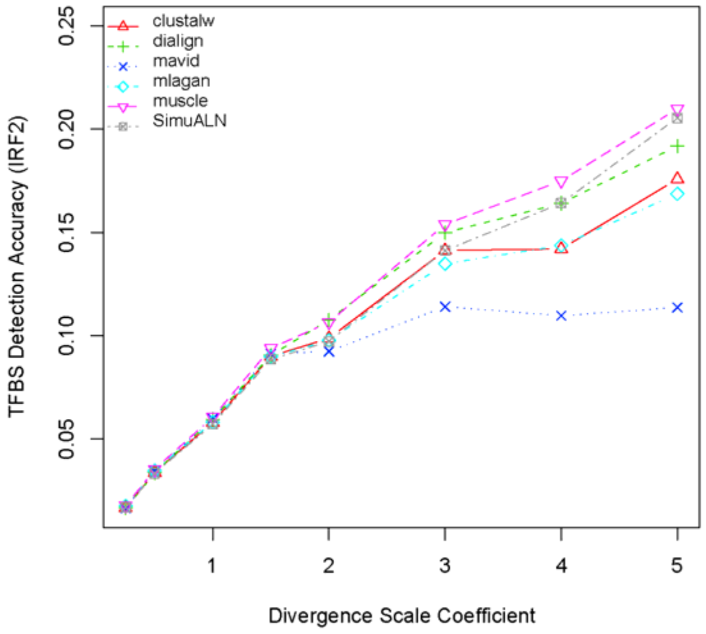

Figure 13: Comparison of the detection accuracy of IRF2 turnover sites

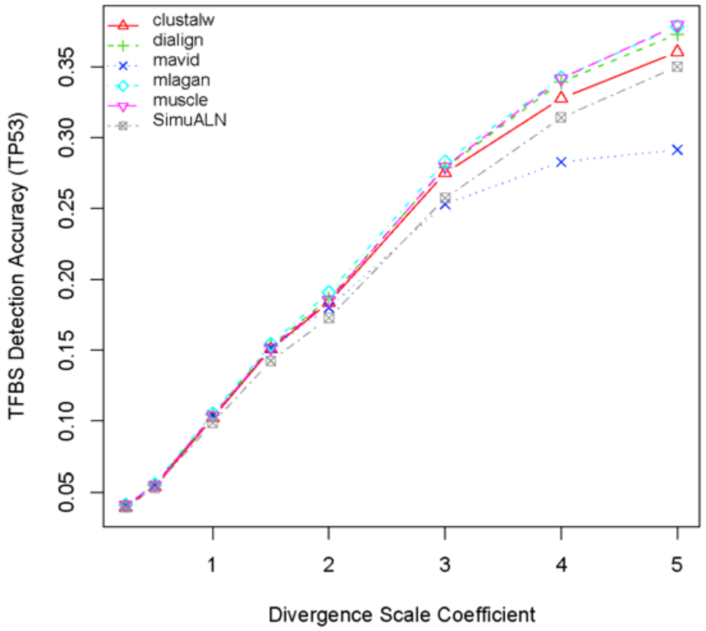

Figure 14: Comparison of the detection accuracy of TP53 turnover sites

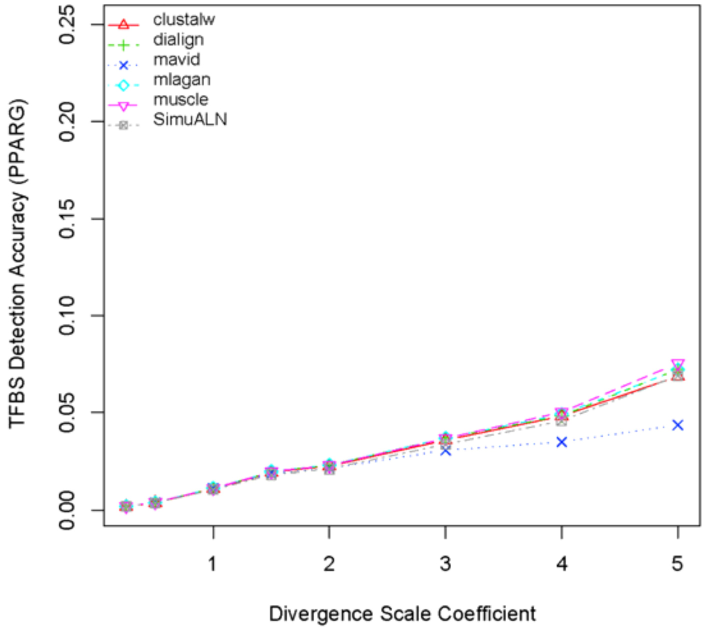

Figure 15: Comparison of the detection accuracy of PPARG turnover sites

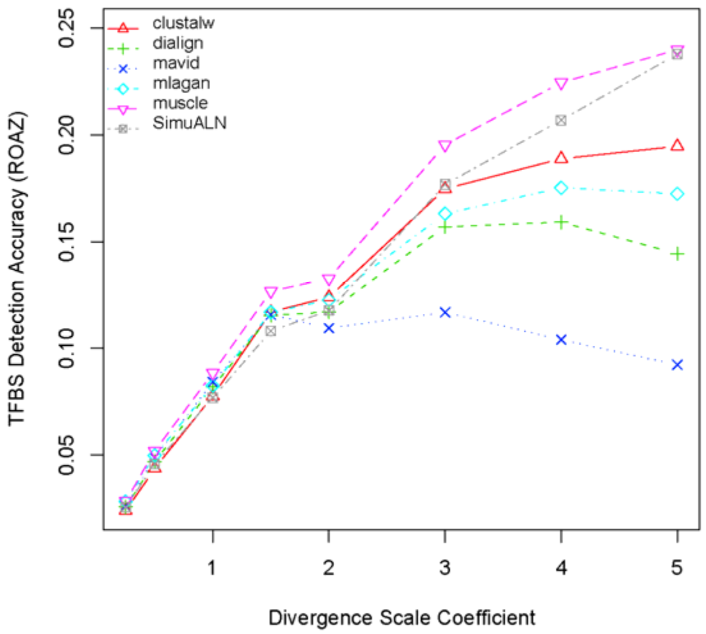

Figure 16: Comparison of the detection accuracy of ROAZ turnover sites
